# The novel GINS4 axis promotes gastric cancer growth and progression by activating Rac1 and CDC42

**DOI:** 10.7150/thno.36256

**Published:** 2019-10-21

**Authors:** Zhonglin Zhu, Zhilong Yu, Zeyin Rong, Zai Luo, Jing Zhang, Zhengjun Qiu, Chen Huang

**Affiliations:** Department of General Surgery, Shanghai General Hospital, Shanghai Jiaotong University School of Medicine, Shanghai, China.

**Keywords:** Gastric cancer, GINS4, Rac1 and CDC42, CircMLLT10, Growth and progression.

## Abstract

**Rationale**: As a component of GINS complex, GINS4 is essential for initiating DNA replication and elongation of the cell cycle G1/S phase in eukaryotes and plays a vital role in normal physiological processes. However, the precise functions and regulation mechanisms of GINS4 in human tumors remain elusive.

**Methods**: GINS4 expression was analyzed in gastric cancer tissues by qRT-PCR and western blotting, and its clinical relevance was studied using TMA. The biological functions of GINS4 were detected *in vitro* and* in vivo*. cDNA array, co-IP, GST pull-down and GTPase activation assays were performed to investigate the downstream regulation mechanism of GINS4. Upstream regulation mechanism of GINS4 was explored and demonstrated by circRNA sequencing, bioinformatics analysis, luciferase reporter assay and rescue experiments.

**Results**: Strikingly high GINS4 expression was detected in gastric cancer tissues and correlated with poor differentiation, advanced tumor stage, invasion depth and lymph node metastasis. GINS4 promoted cell growth and metastasis* in vitro* and *in vivo*, and suppressed cell apoptosis *in vitro*. Mechanistically, GINS4 activated Rac1/CDC42 through directly binding to Rac1/CDC42, thereby activating their downstream pathways. Furthermore, circMLLT10 acts as a miR-509-3-5p sponge to attenuate its repressive effect on target GINS4. In addition, circMLLT10 promoted cell growth and metastasis and suppressed cell apoptosis, whereas miR-509-3-5p inhibited cell growth and metastasis and promoted cell apoptosis.

**Conclusion**: The findings indicate for the first time that the novel GINS4 axis promotes gastric cancer cell growth and progression by activating Rac1 and CDC42. GINS4 may be a promising biomarker and target for diagnosis and treatment of gastric cancer.

## Introduction

Gastric cancer is the fifth most common cancer and the third major cause of cancer-related deaths worldwide 1. In China, gastric cancer was estimated to have the second highest morbidity and mortality rates among all cancers, with 679,100 new cases and 498,000 mortalities in 2015^2^. Despite of great progress in surgery, radiotherapy and chemotherapy for gastric cancer, the overall 5-year survival rate for patients in advanced stages is still less than 30% 3. Hence, elucidating the molecular mechanisms underlying gastric cancer development and progression is extremely urgent 4, 5, as is exploring novel targets for early diagnosis and treatment.

In the current study, we sought to identify crucial genes associated with gastric cancer development and progression by analyzing the Cancer Genome Atlas (TCGA) database. There were 443 gastric cancer samples with available data, including 416 samples with mRNA chip or RNA-seq data, of which 32 paired samples had associated RNA-seq v2 data and pathological data. Differentially expressed genes between above 32 paired samples were screened, and we found that GINS4 expression was significantly higher in gastric cancer tissues than in paired normal gastric tissues (3.74-fold change, P<0.001).

The GINS complex was first identified by Takayama in budding yeast and was proven to be essential for the initiation and elongation of chromosome replication 6. The GINS complex, a heterotetrameric structure comprising four different proteins (Psf1, Psf2, Psf3 and GINS4), interacts with CDC45 and MCM2-7, which is referred to as the “CMG complex”^7^, ^8^. During the G1 phase of the cell cycle, the well-conserved origin recognition complex (ORC) binds to replication origins. ORC is then bound by CDC6, recruits the CMG complex and CDCl0-dependent transcript 1 (CDT1), and ultimately forms the pre-replicative complex (pre-RC), functioning as a replicative helicase to initiate and elongate the replication fork in S phase 9, 10. As an indispensable component of the GINS complex, GINS4, also named Sld5, plays a pivotal role in the progression of DNA replication initiation and elongation 11. In a previous study, we found ectopic expression of GINS4 in gastric cancer tissues and differential expression in various stages of gastric cancer. However, only one published study has reported the biological functions of GINS4 in human cancers 12. Accordingly, the precise biological functions and molecular mechanisms of GINS4 in gastric cancer growth and progression remain unclear, and the mechanisms by which GINS4 expression is modulated remains elusive.

Circular RNAs (circRNAs), recently discovered as a novel type of non-coding RNA, have attracted great attention in genomic research 13. An increasing amount of evidence indicates that circRNAs may be involved in the progression of various tumors, such as colon cancer, oral squamous cell carcinoma (OSCC), hepatocellular carcinoma, bladder cancer and breast cancer 14-18. In 2011, Professor Pandolfi et al. proposed a competing endogenous RNA (ceRNA) hypothesis, whereby mRNA, lncRNA and pseudogenes competitively bind to microRNA response elements (MREs) to regulate downstream mRNA expression 19. Two papers published in *Nature* in 2013 determined that CDR1as (also named ciRS-7) and circular* Sry* RNA are ceRNAs 20, 21. However, the biological functions and detailed mechanisms of circRNAs in gastric cancer progression are less well understood. In this study, a novel circMLLT10/miR-509-3-5p/GINS4/Rac1/CDC42 axis was discovered. This finding is expected to offer promising diagnostic and therapeutic targets for gastric cancer.

## Methods

### Patients and clinical specimens

From 2013 to 2014, 57 paired gastric cancer and adjacent normal samples were obtained from Shanghai General Hospital and immediately fixed in formalin. The samples were embedded in paraffin for tissue microarray (TMA) construction, and the final TMA contained 54 paired gastric cancer samples. From 2015 to 2016, fresh specimens (61 pairs) were collected from patients with primary gastric cancer during surgical resection. All clinicopathological diagnoses were confirmed by two pathologists according to the guidelines of the Union for International Cancer Control (UICC). None of the patients received radiotherapy or chemotherapy at any time before surgery. Before enrollment in this study, written informed consent was obtained from all subjects. The project was approved by the Ethics Committee of Shanghai General Hospital.

### Cell lines and culture conditions

Human gastric cancer cell lines (HGC-27, MKN-28, MKN-45, MGC-803, BGC-823, AGS, SGC-7901) and 293T cells were purchased from Type Culture Collection of the Chinese Academy of Science (Shanghai, China). All cell lines above were cultured in RPMI 1640 medium with 10% fetal bovine serum (FBS) (Gibco, Grand Island, NY, USA) and 1% penicillin-streptomycin at 37 °C in a humidified atmosphere containing 5% CO_2_.

### Animal experiments

To study primary gastric tumor growth, 20 male BALB/c athymic nude mice (4 weeks old) were randomly divided into 4 groups (n=5), and injected subcutaneously into subscaples with 1.0×10^7^ stable gastric cells to establish the gastric cancer xenograft model. Tumor size was measured twice a week to monitor tumor growth. Both the minimum (W) and maximum (L) diameters were measured for all tumors, and the volume was computed as πLW^2^/6. All mice were sacrificed after 4 weeks, and the tumors were surgically removed and weighed.

To explore the effects of GINS4 on metastasis of cancer, we established two types of mouse models: the liver metastasis and the peritoneal metastasis. For the liver metastasis models, 1.0×10^7^ cells were intravenously injected into ileocolic vein of nude mice as we did previously 22. For peritoneal metastasis models, 1.0×10^7^ stable cells were injected into the abdominal cavity of nude mice. After 4 weeks, all mice were sacrificed. Finally, the livers were removed and validated by hematoxylin and eosin (H&E) staining. The peritoneal metastatic nodules were observed and counted. All animal experiments were administered under the guidelines for the care and use of laboratory animals and were approved by the Institutional Animal Care and Use Committee of Shanghai General Hospital.

### *In situ* hybridization (ISH)

After de-waxing and re-hydration, the TMA was treated with Proteinase K for 10 min at 37 °C. Next, the TMA was incubated with hybridization mix for 1 h at 57 °C, followed by washing with hydrophobic barrier. The TMA was then blocked for 15 min in blocking solution and hybridized with digoxigenin (DIG)-labeled miR-509-3-5p probes at 50 °C for 16 h. After washing twice, the TMA was treated with 0.5% blocking reagent for 30 min and incubated with anti-DIG and horseradish peroxidase for 2 h at room temperature. After washing twice with TBST and dehydration with xylene, the TMA was covered with coverslips. The staining scores were evaluated by two pathologists.

### Luciferase reporter assay

For this assay, luciferase plasmids were co-transfected into cells with miR-509-3-5p mimics or inhibitors using Lipofectamine^TM^ 2000 reagent. After 36 h, the cells were washed with pre-cold PBS, lysed with 100 μl of 1×passive lysis buffer (PLB, Promega, USA) and then incubated at room temperature for 15 min with rocking. The cell suspensions (20 μl) and 100 μl of LARII were added into luminometer tubes, followed by firefly luciferase activity detection. Renilla luciferase activity was detected after addition of 100 μl of Stop & GloR reagent. Plasmid activity was calculated as the ratio of firefly luciferase/Renilla luciferase activity.

### Co-immunoprecipitation assay (co-IP)

Briefly, 293T cells were transfected with Flag-GINS4 or Flag-vector plasmids for 24 h. Cells were treated with lysis buffer (20 mM Tris-HCl (pH 8), 137 mM NaCl, 0.5% Triton X-100, 2 mM EDTA) and protease inhibitor cocktail (Sangon Biotech, Shanghai, China) at 4 °C for 30 min. The lysates were centrifuged and separated at 14,000×g for 15 min. For co-IP assays, 500 μg of proteins were prepared and incubated with 20 µl of an anti-FLAG-M2 agarose slurry (Sigma, USA). Then, the beads were washed three times with lysis buffer, centrifuged and collected at 500×g for 5 min. The precipitated proteins were eluted in 1×SDS-PAGE loading buffer and boiled for 10 min. Western blotting was performed using the precipitated proteins and cell lysates.

### Glutathione S-transferase (GST) pull-down assay

GST and GST fusion proteins (GST-Rac1, GST-CDC42) were constructed using *Escherichia. coli* BL21 cells and were purified with glutathione Sepharose 4B beads. The His-GINS4 fusion protein was expressed in BL21 Rosetta (*DE3*) cells and was purified and collected with Ni-NTA beads. His-GINS4 protein was rotated with GST, GST-Rac1 or GST-CDC42 at 4 °C for 4 h and then added to Ni-NTA beads for an additional 4 h at 4 °C. After centrifugation and three washes, the beads were eluted with 30 μl of 1×SDS-PAGE loading buffer and then boiled for 10 min, followed by western blotting.

### GTPase activation assay

Cells were harvested in ice-cold cell lysis buffer and centrifuged at 16000 × g at 4 °C for 5 min. Proteins (120 μg in 600 μl) were added into two tubes with 1/10 volume loading buffer. Then, 1/100 volume GDP was added into one tube, and 1/100 volume GTPγS was added into the other tube. The two tubes were incubated at room temperature for 15 min; then, the reaction was stopped with the addition of stop buffer. Each tube was added with 20 μg of PAK-GST protein beads, and rotated at 4 °C for 1 h. After centrifugation at 5200×g at 4 °C for 1 h, the beads were washed twice. GTP-Rac1 and GTP-CDC42 were eluted with 25 μl of SDS loading buffer. Then, the proteins of samples were analyzed by western blotting.

### Fluorescence *in situ* hybridization (FISH) and confocal laser scanning microscopy

FISH was performed to determine the location of circMLLT10 using a FAM-labeled probe (5'-CTGTTATAAATACTGGTGTGAGCTG-3') and of miR-509-3-5p using a Cy3-labeled probe (5'-CATGATTGCCACGTCTGCAGTA-3'). MGC-803 and AGS cells were seeded in 35-mm glass bottom dishes with 10-mm microwells. After washing in PBS and fixing with anhydrous ethanol, the cells were treated with 100 μl of 0.1% Triton-100 at room temperature for 15 min. After washing and dehydrating, the cells were hybridized overnight at 37 °C with 5 µl of probe in hybridization buffer (10% dextran sulfate, 40% formamide, 4× saline-sodium citrate, 1× Denhardt's solution, 1000 mg/ml sheared salmon sperm DNA, 10 mM DDT, 1000 mg/ml yeast transfer RNA). The next day, the cells were continuously washed and stained with 100 μl of DAPI for 20 min. Confocal laser scanning microscopy was then used to observe the staining.

### Statistical analysis

All quantitative data were calculated by the χ^2^ or Fisher's exact test. The correlations were analyzed by Pearson's test (r, P). Paired and unpaired continuous variables were compared by Student's t-test or the Mann-Whitney U test. The survival curves were drawn using the Kaplan-Meier method and COX multivariate analysis, and were analyzed by log-rank tests. P<0.05 was considered statistically significant in all tests. The SPSS 22.0 software was conducted for statistical analyses.

Details about cell culture conditions, transfection, ingenuity pathway analysis (IPA), cell counting assays, MTT assays, flow cytometry, cell wound healing and transwell assays, real-time PCR, western blotting and immunohistochemistry were described in Additional file 3: Supplementary materials and methods.

## Results

### GINS4 expression in gastric cancer tissues and cell lines

**Figure 1:**
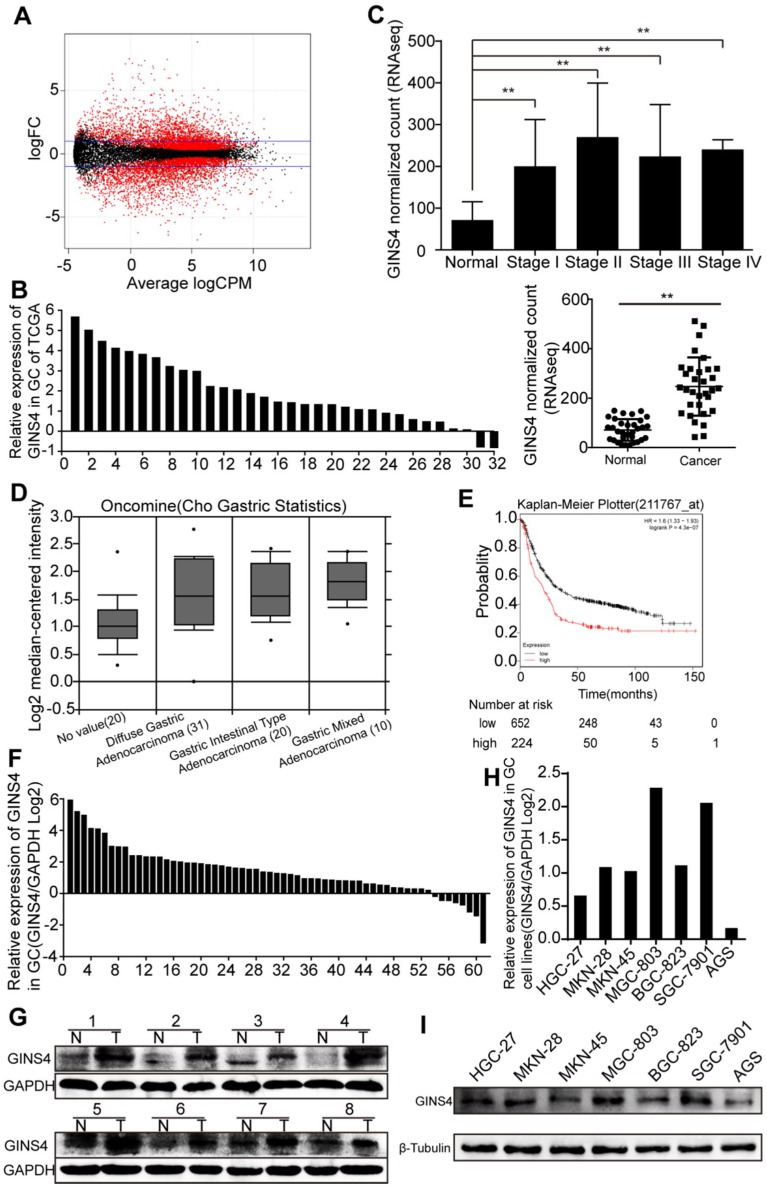
** GINS4 expressions in gastric cancer tissues and cell lines. A**, The scatter diagram shows the differential mRNA expression among 32 gastric cancer samples and paired adjacent normal gastric samples in TCGA database. **B**, GINS4 mRNA expression in 32 paired gastric cancer tissues. **C**, GINS4 expression level in stages I, II, III and IV mucosae compared with adjacent normal mucosa. **D**, GINS4 expression in gastric cancer specimens from the Oncomine dataset (Cho gastric statistics, 2011). **E**, OS data for gastric cancer patients from the Kaplan-Meier Plotter analysis. **F**, GINS4 mRNA expression in 61 fresh frozen gastric cancer tissues. **G**, GINS4 protein expression in 8 paired gastric cancer tissues. **H&I**, GINS4 mRNA and protein expression in 7 gastric cancer cell lines.

Differentially expressed genes in 32 paired gastric cancer samples from TCGA database were analyzed (**Figure 1A**), and GINS4 expression was found to be significantly higher in gastric cancer tissues than the adjacent normal tissues (**Figure 1B&C**). We also found that GINS4 expression was increased in 61 gastric cancer tissues (Cho gastric statistics, 2011) from the Oncomine database (**Figure 1D**). In addition, the Kaplan-Meier Plotter analysis revealed that overall survival (OS) was substantially lower in gastric cancer patients with high GINS4 expression than in those with low GINS4 expression (**Figure 1E**). To further evaluate GINS4 expression in gastric cancer tissues, qRT-PCR and western blotting were performed, and GINS4 expression was markedly increased in gastric cancer tissues (**Figure 1F&G**). GINS4 mRNA and protein expression was also detected in 7 gastric cancer cell lines, with MGC-803 cells presenting the highest GINS4 expression, and AGS cells the lowest GINS4 expression (**Figure 1H&I**). Therefore, MGC-803 and AGS cells were selected for further analyses.

### GINS4 expression correlated closely with gastric cancer clinicopathological features

**Figure 2:**
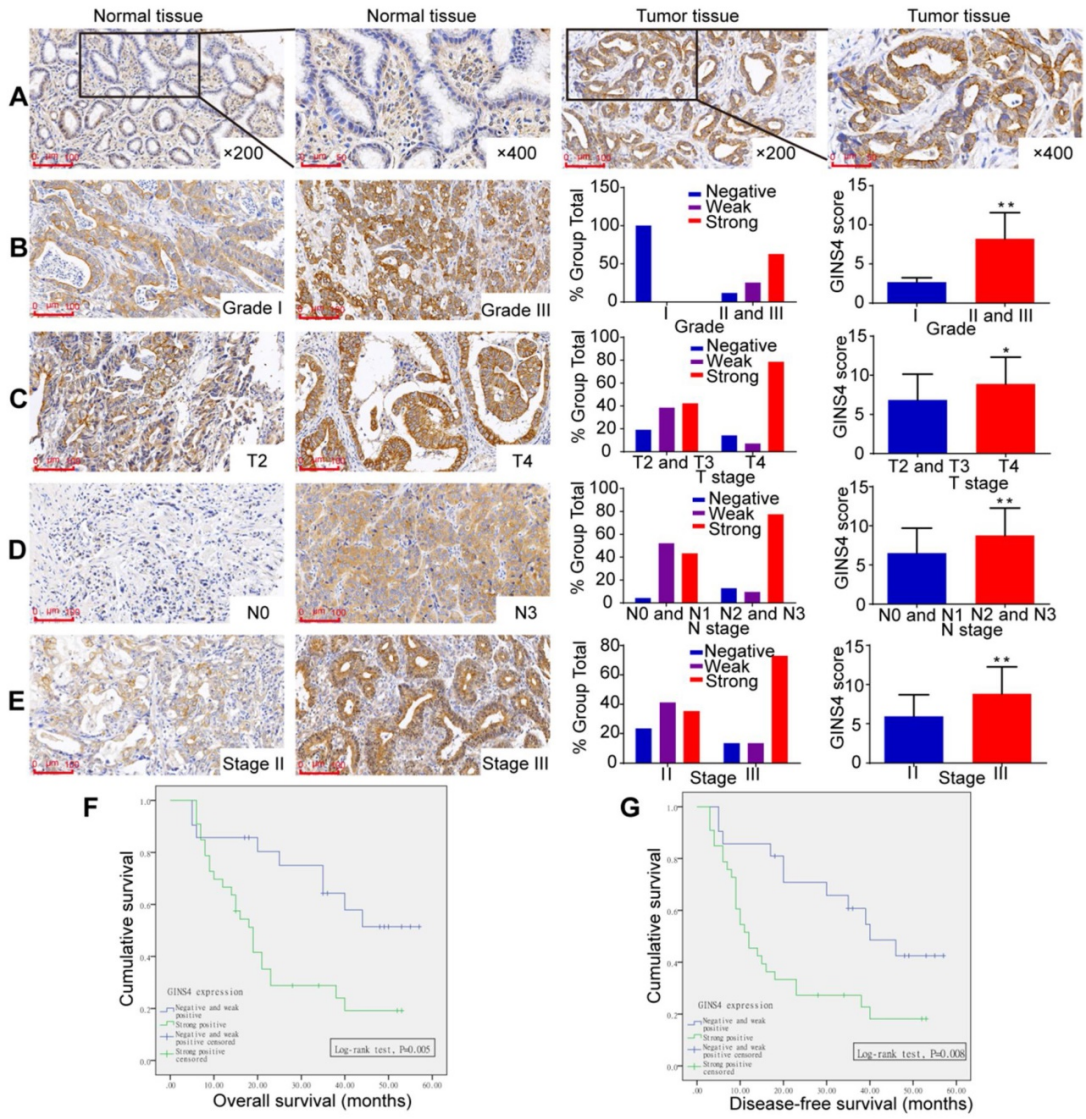
** GINS4 expression in the TMA and its correlation with clinicopathological features.** The TMA, including 54 gastric cancer tissues and matched normal tissues, was stained with a specific anti-GINS4 antibody. **A**, Representative images of GINS4 expression in normal gastric tissues and matched gastric cancer tissues (200×, 400×). **B-E**, GINS4 expression between grade I *vs* grades II and III (**B**, *P*<0.01), T4 *vs* T2 and T3 (**C**, *P*<0.05), N0 and N1 *vs* N2 and N3 (**D**, *P*<0.01), and stage II *vs* stage III (**E**, *P*<0.01). **F&G**, The OS and DFS of patients with strong positive GINS4 expression *vs* those with negative/weak positive GINS4 expression. *P<0.05, **P<0.01.

GINS4 expression was investigated by TMA. GINS4 expression was significantly higher in gastric cancer tissues than in adjacent normal gastric tissues (**Figure 2A, Table 1**). Moreover, increased GINS4 expression was correlated with worse tumor differentiation, and a significant difference between grade I (well) *vs* grades II and III (moderate and poor) was found (**Figure 1B, Table 1,** *P*=0.003). Additionally, GINS4 expression was positively correlated with T stage (T2 and T3 *vs* T4, **Figure 2C, Table 1**, *P*=0.008), N stage (N0 and N1 *vs* N2 and N3, **Figure 2D**, **Table 1**, *P*=0.002) and disease stage (stage II *vs* stage III, **Figure 2E**, **Table 1**, *P*=0.024). Univariate survival analysis revealed that patients with strong GINS4-positive staining in surgically obtained tissues had low OS and disease-free survival (DFS) (**Figure 2F&G**). Additionally, T, N, and UICC stages and GINS4 expression were closely correlated with OS and DFS (**Table 2**). According to multivariate survival analysis, no factors were correlated with OS and DFS (**Table 2**). These findings indicate that GINS4 plays a pivotal role in gastric cancer development and progression and may function as a promising target for gastric cancer therapy and diagnosis.

### GINS4 promoted gastric cancer cell growth and metastasis *in vitro*

**Figure 3:**
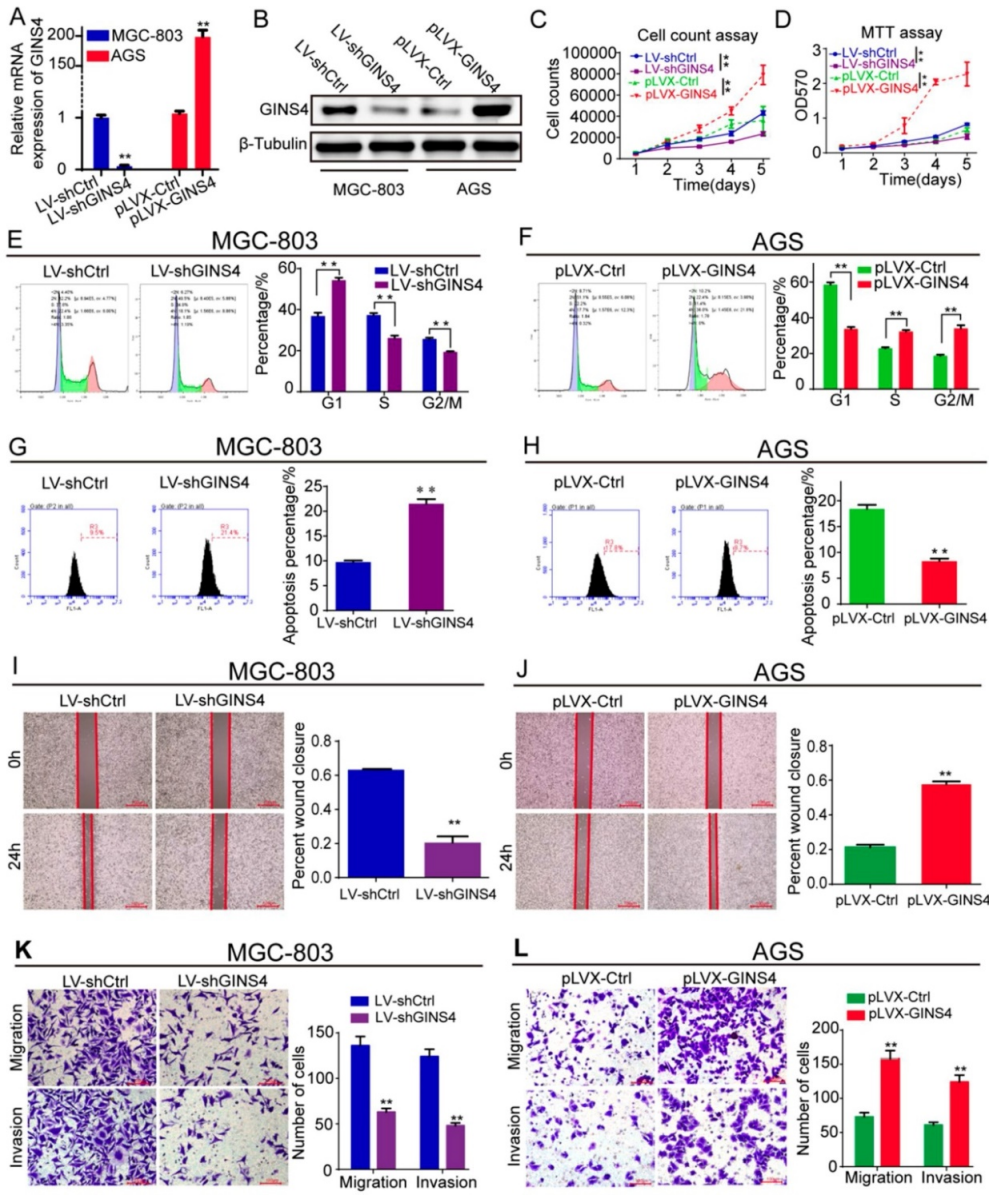
** GINS4 promotes cell proliferation, cell cycle, migration and invasion, and suppresses cell apoptosis *in vitro*.** Stable MGC-803 cells with GINS4 knockdown and stable AGS cells with GINS4 overexpression were constructed. **A&B**, GINS4 mRNA and protein expression in stable MGC-803 and AGS cells were detected. **C**, Cell counting of MGC-803 and AGS cells with different GINS4 levels. **D**, MTT assays of MGC-803 and AGS cells with different GINS4 levels. **E&F**, Flow cytometric analysis of the cell cycle in MGC-803 and AGS cells. **G&H**, Flow cytometric analysis of cell apoptosis in MGC-803 and AGS cells. **I&J**, Cell wound healing assays of MGC-803 and AGS cells with different levels of GINS4.** K&L,** Transwell migration and invasion assays of MGC-803 and AGS cells with different levels of GINS4. All data are presented as the mean ± SEM of three experiments. *P<0.05, **P< 0.01.

To determine the biological functions of GINS4 in gastric cancer, LV-shGINS4 and pLVX-GINS4 vectors were constructed, and their effects on GINS4 expression were verified by qRT-PCR and western blotting (**Figure 3A&B**). Cell counting and MTT assays revealed that the down-regulation of GINS4 expression notably decreased the proliferation rate of MGC-803 cells, whereas the up-regulation of GINS4 expression markedly increased that of AGS cells (**Figure 3C&D**). Regarding to the cell cycle, knockdown of GINS4 expression increased G1 phase arrest, and enhanced GINS4 expression promoted the G1/S phase transition (**Figure 3E&F**). For cell apoptosis assay, down-regulation of GINS4 expression promoted apoptosis in MGC-803 cells, whereas up-regulated GINS4 expression inhibited this process in AGS cells (**Figure 3G&H**). Furthermore, cell wound healing and transwell migration and invasion assays indicated that knockdown of GINS4 decreased the ability of mobility, migration and invasion (**Figure 3I&K**), while overexpression of GINS4 enhanced the ability of mobility, migration and invasion (**Figure 3J&L**). Taken together, these results demonstrated that GINS4 promotes cell proliferation, cell cycle and metastasis, and suppresses cell apoptosis in gastric cancer.

### GINS4 promoted gastric cancer cell growth and metastasis *in vivo*

**Figure 4:**
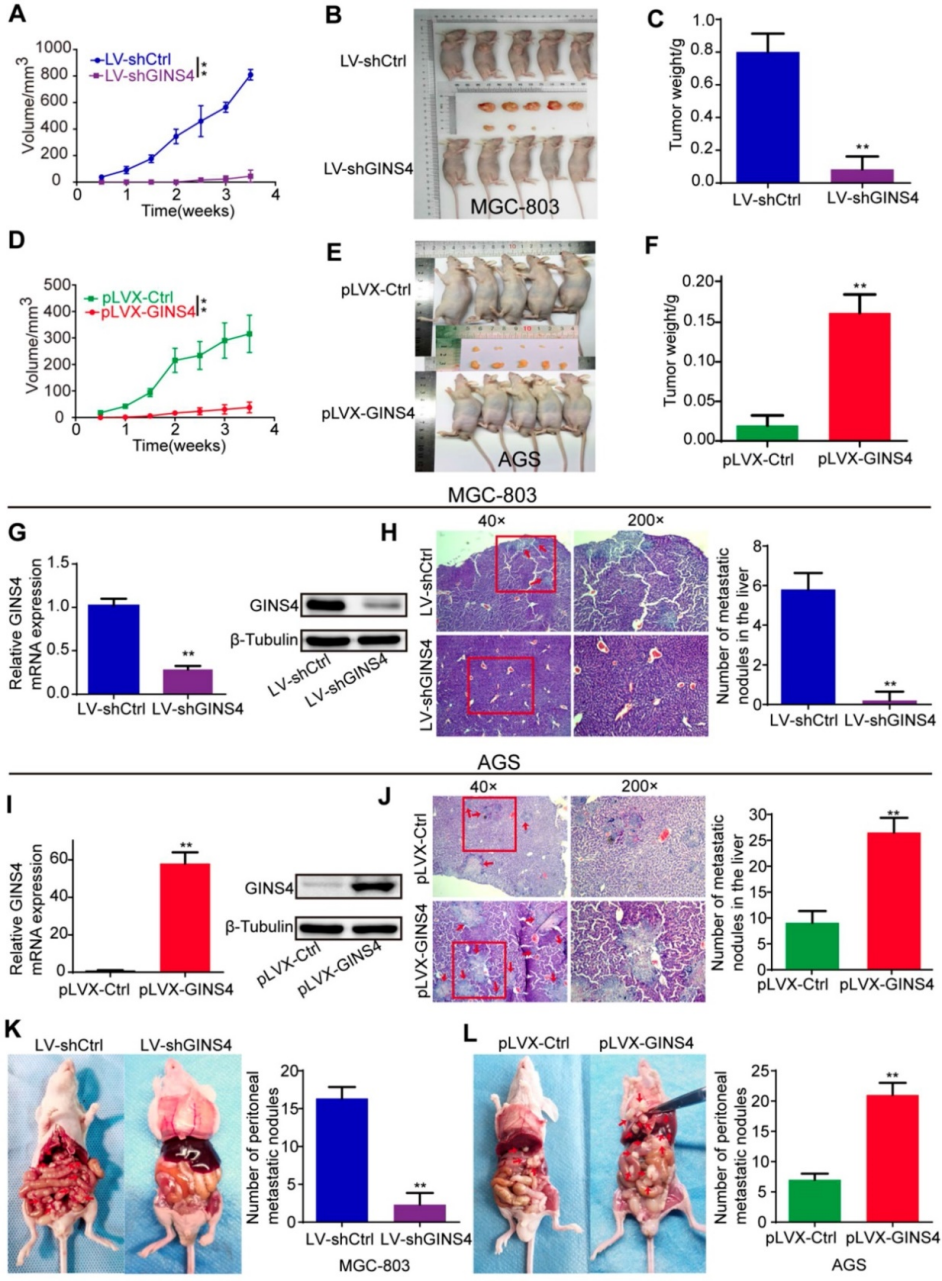
** GINS4 promotes cell growth and metastasis of gastric cancer *in vivo*. A-F,** a nude mouse xenograft model was constructed. **A,** Tumor volumes of MGC-803 cells were measured 2 times every week for 4 weeks. **B**, Images of subcutaneous xenograft tumors of MGC-803 cells. **C,** The final tumor weight of MGC-803 cells was shown. **D**, Tumor volumes of AGS cells were measured 2 times every week for 4 weeks. **E,** Images of subcutaneous xenograft tumors of AGS cells. **F,** The final tumor weight of AGS cells was shown. **G**, The expression of GINS4 mRNA and protein in tumors with MGC-803/LV-shGINS4 was significantly lower than that in tumors with MGC-803/LV-shCtrl. **H,** Representative images of HE staining of the liver metastasis. Knockdown of GINS4 decreased the number of the liver metastatic nodules compared with negative control. **I,** The expression of GINS4 mRNA and protein in tumors with AGS/pLVX-GINS4 was significantly higher compared with that in tumors with AGS/pLVX-Ctrl. **J,** Representative images of HE staining of the liver metastasis. Overexpression of GINS4 increased the number of the liver metastatic nodules compared with negative control. **K&L,** Representative images of peritoneal metastasis assays. Knockdown of GINS4 decreased the number of peritoneal metastatic nodules (**K**), whereas overexpression of GINS4 increased the number of peritoneal metastatic nodules (L) **P < 0.01.

To explore the roles of GINS4 in cell growth *in vivo*, a nude mouse xenograft model of gastric cancer was constructed. Tumor volume was monitored twice a week. Tumor growth was found to be significantly inhibited by knockdown of GINS4 but enhanced by overexpression of GINS4 (**Figure 4A&D**). Meanwhile, the sizes and weights of the MGC-803/LV-shGINS4 tumors were significantly lower than those of the MGC-803/LV-shCtrl tumors (**Figure 4B&C**), and the AGS/pLVX-GINS4 tumors were larger than the AGS/pLVX-Ctrl tumors (**Figure 4E&F**). In addition, we confirmed that the level of GINS4 was obviously lower in tumors with MGC-803/LV-shGINS4 than those of the MGC-803/LV-shCtrl (**Figure 4G**), while higher in AGS/pLVX-GINS4 tumors than the AGS/pLVX-Ctrl tumors (**Figure 4I**).

The role of GINS4 on metastasis was confirmed by the liver metastasis and the peritoneal metastasis models *in vivo*. In the liver metastasis models, necropsy and HE staining showed that the numbers of liver metastatic nodules were less in mice bearing MGC-803/LV-shGINS4 cells compared with MGC-803/LV-shCtrl (**Figure 4H**), whereas the numbers were higher in mice bearing AGS/pLVX-GINS4 cells than the AGS/pLVX-Ctrl cells (**Figure 4J**). In peritoneal metastasis models, we observed that the number of peritoneal metastatic nodules was less in mice bearing MGC-803/LV-shGINS4 cells compared with MGC-803/LV-shCtrl (**Figure 4K**), while AGS/pLVX-GINS4 cells colonized the visceral organs and formed multiple metastatic nodules (**Figure 4L**), indicating that GINS4 enhances tumor colonization and peritoneal metastasis. Collectively, our results demonstrate that GINS4 plays an oncogenic role in gastric cancer, and promotes cancer growth and progression *in vivo*.

### cDNA array and IPA results revealed that GINS4 promotes gastric cancer growth and progression through the Rac1/CDC42 pathway

**Figure 5:**
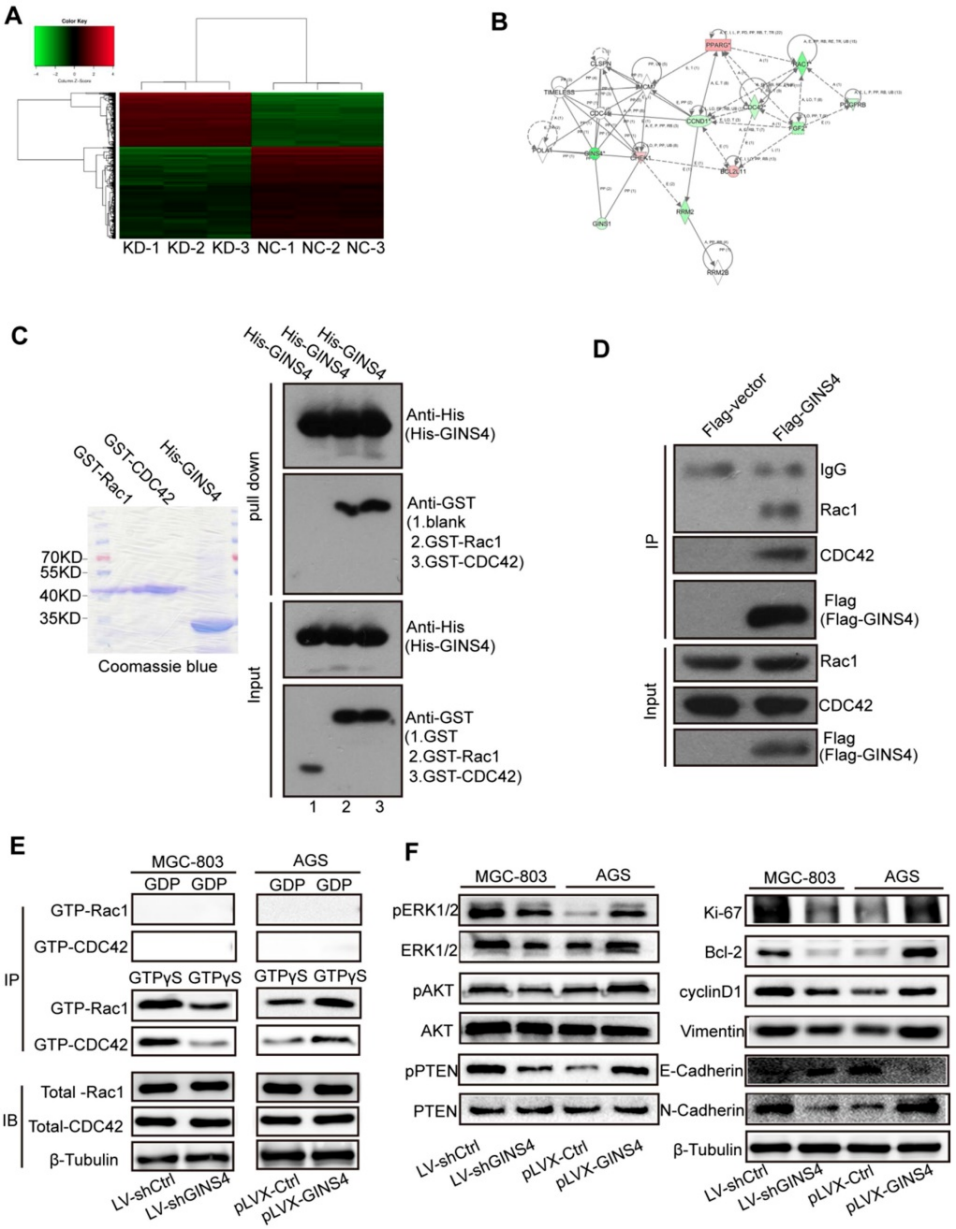
** GINS4 activates the Rac1/CDC42 and their downstream pathways. A**, The human cDNA microarray for differentially expressed mRNA between MGC-803/LV-shGINS4 (KD-1, 2, 3) and MGC-803/LV-shCtrl cells (NC-1, 2, 3). **B**, IPA revealed the interaction network of GINS4 in gastric cancer growth and metastasis. **C,** GST pull-down assay showed that exogenous Rac1 and CDC42 were pulled down by the His-GINS4 protein and detected using an anti-GST antibody. **D,** For co-IP assay, flag-vector or Flag-GINS4 plasmids were transfected into 293T cells. Endogenous Rac1 and CDC42 were immunoprecipitated with an anti-Flag antibody. **E**, GTPase activation assays showed that knockdown of GINS4 decreased the level of GTP-Rac1 and GTP-CDC42, while overexpression of GINS4 increased the level of GTP-Rac1 and GTP-CDC42.** F**, Altered GINS4 expression changed the expression of pERK1/2, pAKT and pPTEN, also the expression of cell proliferation-, cycle-, apoptosis- and metastasis-related proteins (Ki-67, Bcl-2, cyclinD1, E-Cadherin, N-Cadherin and Vimentin). *P<0.05, **P<0.01.

To further investigate the underlying molecular mechanisms of GINS4, a cDNA array (**Figure 5A**) was performed to identify differentially expressed genes between MGC-803/LV-shGINS4 and MGC-803/LV-shCtrl cells. Among the 1065 differentially expressed genes with >2.0- or <0.5-fold changes (*P*<0.05), 403 genes were up-regulated, and 662 genes down-regulated, with BCL2L11, cyclinD1, CDC42, PDGFRB, RAC1, FGF2 and SKP2 exhibiting the lowest expression levels. Furthermore, the IPA results indicated that GINS4 is implicated in cancer cell proliferation, cell cycle, apoptosis and metastasis through Rac1 and CDC42 and their downstream signaling pathways: MAPK/ERK pathway, PI3K/AKT pathway and PTEN pathway (**Figure 5B, Figure S1**).

Rac1 and CDC42 expression were detected by IHC with the TMA of gastric cancer tissues. The clinical relevance of Rac1 and CDC42 is shown in **Figure S2**, **Table S1-2**.

### GINS4 directly bound to and activated Rac1/CDC42

To further verify the regulatory mechanism of GINS4 on Rac1/CDC42, GST pull-down, co-IP, and GTPase activation assays were performed. In GST pull-down assay, exogenous GST, GST-Rac1, GST-CDC42 and His-GINS4 were cloned and purified (**Figure 5C**), and His-GINS4 beads were used to pull-down potential GST-Rac1 and GST-CDC42 protein. *In vitro* experiments demonstrated that both GST-Rac1 and GST-CDC42 proteins were pulled down by exogenous GINS4 (**Figure 5C**), which indicated that GINS4 interacts with Rac1 and CDC42. In the co-IP assay of 293T cells (**Figure 5D**) and MGC-803 cells (**Figure S3A**), endogenous Rac1 and CDC42 proteins were immunoprecipitated by the Flag-GINS4 protein, further demonstrating the direct binding between GINS4 with Rac1 and CDC42. Therefore, co-IP and GST pull-down assays demonstrated that GINS4 directly binds to Rac1 and CDC42. As we all know, among the total Rac1 and CDC42, GTP-Rac1 and GTP-CDC42 possess biological activity, whereas GDP-Rac1 and GDP-CDC42 could not exert functions. To further explore the effects of GINS4 on Rac1 and CDC42 activities, PAK-GST protein beads were used to pull-down GTP-Rac1 and GTP-CDC42. The results revealed that knockdown of GINS4 in MGC-803 cells reduced the level of GTP-Rac1 and GTP-CDC42, while overexpression of GINS4 in AGS cells increased the level of GTP-Rac1 and GTP-CDC42 (**Figure 5E**). Furthermore, we detected the effects of GINS4 on the downstream pathway of Rac1 and CDC42. Knockdown of GINS4 decreased the expression of pERK1/2, pAKT and pPTEN, also the expression of cell proliferation-, cycle-, apoptosis- and metastasis-related proteins (Ki-67, Bcl-2, cyclinD1, N-Cadherin and Vimentin); while overexpression of GINS4 increased the expression of pERK1/2, pAKT and pPTEN, also the expression of cell proliferation-, cycle-, apoptosis- and metastasis-related proteins; (**Figure 5F**) indicating that GINS4 affects cell proliferation, cell cycle, apoptosis and metastasis through activating MAPK/ERK, PI3K/AKT and PTEN signaling pathways. In summary, GINS4 activates Rac1 and CDC42 through directly binding to Rac1 and CDC42, thereby enhancing MAPK/ERK pathway, PI3K/AKT pathway and PTEN pathways.

### MiR-509-3-5p suppressed GINS4 expression by directly binding to the 3′-untranslated region (3'UTR) of GINS4 mRNA

**Figure 6:**
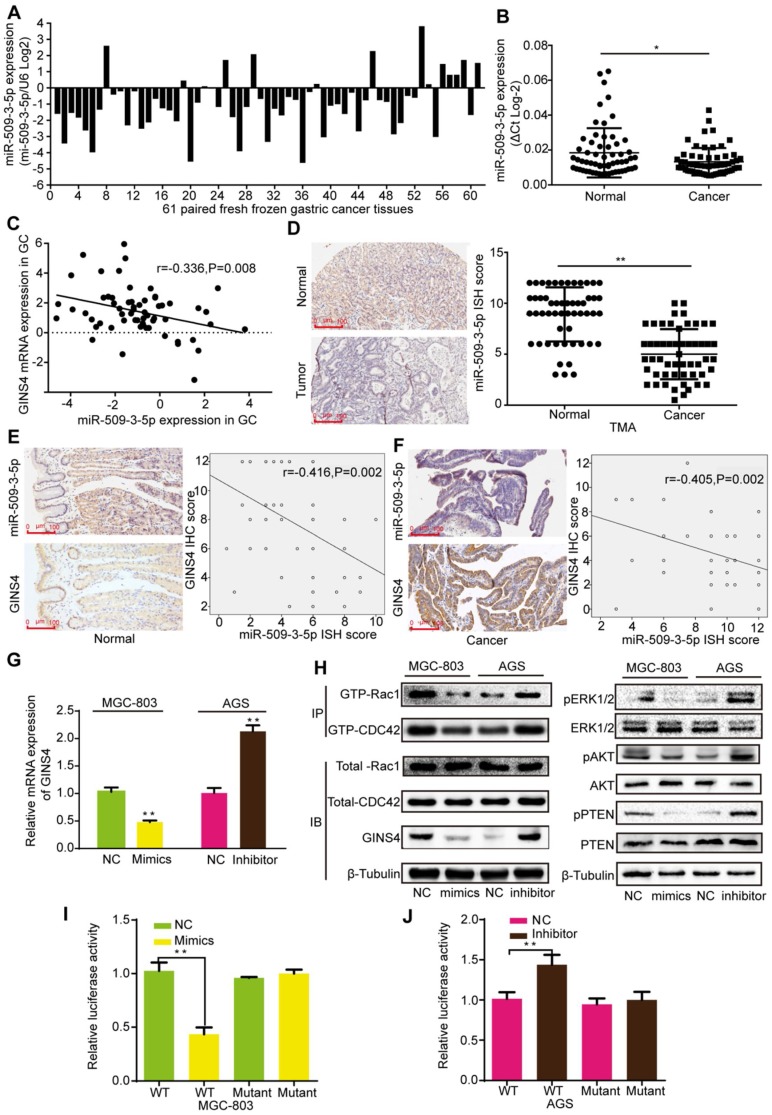
** MiR-509-3-5p suppresses GINS4 expression by directly binding to the GINS4 mRNA 3'UTR. A&B**, MiR-509-3-5p expression in 61 fresh frozen gastric cancer tissues. **C**, The correlation between GINS4 mRNA expression and miR-509-3-5p expression in 61 fresh frozen gastric cancer tissues. **D**, The ISH results showed that the level of miR-509-3-5p was lower in gastric cancer tissues than that in matched adjacent normal tissues of the gastric cancer TMA. **E&F**, The correlation between GINS4 protein expression and miR-509-3-5p expression in normal gastric tissues and gastric cancer tissues of the TMA. **G**, GINS4 mRNA expression was detected after transfection with miR-509-3-5p/mimics or miR-509-3-5p/inhibitor. **H,** The effects of altered miR-509-3-5p expression on the level of GINS4 protein and the activation of Rac1/CDC4 and their downstream pathways were detected. **I&J**, The WT or mutant GINS4 mRNA 3'UTR was respectively cloned into luciferase reporter plasmids. The relative luciferase activity in MGC-803 (**I**) and AGS cells (**J**) is shown. All data are presented as the mean ± SEM of three experiments. *P<0.05, **P< 0.01.

To determine how GINS4 is regulated in gastric cancer, bioinformatics analysis was used to seek for potential upstream miRNAs. MiR-509-3-5p, an anti-oncogenic miRNA, was predicted to bind to the 3'UTR of GINS4 mRNA according to Targetscan, PITA and RNA22 databases. The qRT-PCR results indicated that miR-509-3-5p expression was down-regulated in 77.05% (47/61) of the fresh frozen gastric cancer tissues (**Figure 6A**), and significantly higher in normal gastric tissues than gastric cancer tissues (**Figure 6B**). As determined by Pearson's analysis, miR-509-3-5p expression was negatively correlated with GINS4 mRNA expression in the above gastric cancer tissues (**Figure 6C**). Additionally, the ISH results revealed that miR-509-3-5p expression was significantly lower in gastric cancer tissues than in paired normal gastric tissues (**Figure 6D**). Further analysis of the continuous TMA slices revealed that the miR-509-3-5p ISH scores were negatively correlated with the GINS4 IHC scores in both normal gastric tissues and gastric cancer tissues (**Figure 6E&F**).

To further explore the regulation mechanism, miR-509-3-5p/mimics and miR-509-3-5p/inhibitor were constructed and verified (**Figure S3B**). MiR-509-3-5p/mimics reduced GINS4 mRNA and protein expression in MGC-803 and 293-T cells, whereas miR-509-3-5p/inhibitor increased GINS4 expression in AGS and 293-T cells (**Figure 6G&H, Figure S3C**). To further determine the negative regulation of miR-509-3-5p on GINS4 expression, we analyzed the 3'UTR of GINS4 mRNA, and found a potential binding site for miR-509-3-5p (**Figure S3D**). Thus, the wild type of 3'UTR (WT) and mutant 3'UTR (Mutant) of GINS4 mRNA were cloned into luciferase vectors (**Figure S3D**). MiR-509-3-5p/mimics markedly decreased luciferase activity in the MGC-803 cells carrying WT vectors but not Mutant vectors (**Figure 6I**). In contrast, the miR-509-3-5p/inhibitor increased luciferase activity in the AGS cells with WT vectors but did not affect luciferase activity in those cells with Mutant vectors (**Figure 6J**).

Furthermore, miR-509-3-5p/mimics decreased the level of GTP-Rac1 and GTP-CDC42, also the expression of pERK1/2, pAKT and pPTEN; while miR-509-3-5p/inhibitor increased the level of GTP-Rac1 and GTP-CDC42, also the expression of pERK1/2, pAKT and pPTEN. (**Figure 6H**) Taken together, our results determined that miR-509-3-5p suppresses GINS4 mRNA translation through directly binding to the 3'UTR of GINS4 mRNA, thereby suppressing the activation of Rac1 and CDC42 and their downstream pathways.

### MiR-509-3-5p suppressed the cell growth and metastasis

The possible biological functions of miR-509-3-5p in gastric cancer were investigated. Cell counting and MTT assays showed that miR-509-3-5p/mimics markedly decreased the proliferation rate of MGC-803 cells (**Figure S4A**), while miR-509-3-5p/inhibitor increased the proliferation rate of AGS cells (**Figure S4B**). In cell cycle assays, miR-509-3-5p/mimics induced G1 phase arrest (**Figure S4C**), and miR-509-3-5p/inhibitor promoted G1/S phase transition (**Figure S4D**). Moreover, miR-509-3-5p/mimics promoted cell apoptosis in MGC-803 cells (**Figure S4E**), whereas miR-509-3-5p/inhibitor reduced cell apoptosis in AGS cells (**Figure S4F**). In addition, cell wound healing and transwell migration and invasion assays indicated that miR-509-3-5p/mimics decreased the ability of mobility, migration and invasion (**Figure S4G&I**), while miR-509-3-5p/inhibitor enhanced the ability of mobility, migration and invasion (**Figure S4H&J**). In summary, miR-509-3-5p was proven to suppress cell proliferation, cell cycle and metastasis, and promote cell apoptosis in gastric cancer.

### CircMLLT10 acts as a sponge of miR-509-3-5p

**Figure 7:**
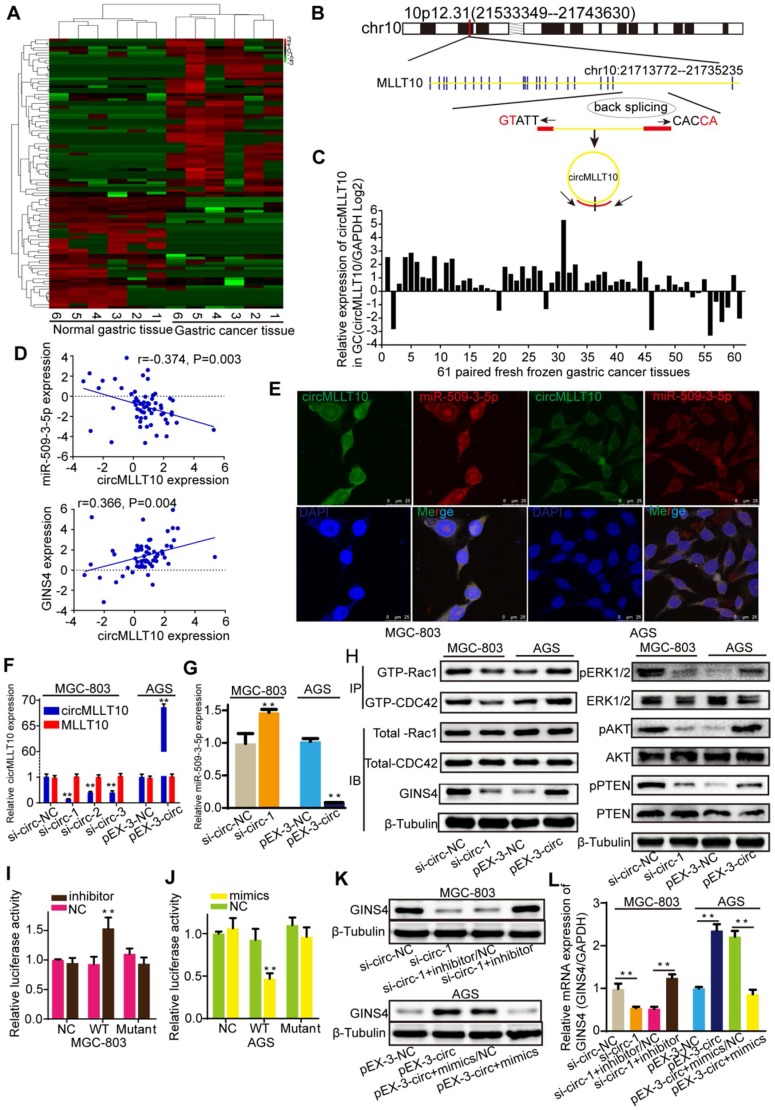
** CircMLLT10 acts as a miRNA sponge and inhibits miR-509-3-5p activity. A**, Heat map of the differentially expressed circRNAs among 6 fresh frozen gastric cancer tissues and paired normal gastric tissues. **B**, Schematic representation of the circMLLT10 production process. **C**, CircMLLT10 expression in 61 fresh frozen gastric cancer tissues. **D**, Pearson's correlation among circMLLT10, miR-509-3-5p, and GINS4 mRNA expression in 61 fresh frozen gastric cancer tissues. **E**, FISH results for circMLLT10 and miR-509-3-5p localization in MGC-803 and AGS cells. **F**, qRT-PCR results for circMLLT10 knockdown in MGC-803 cells and overexpression in AGS cells. **G**, The effect of altered circMLLT10 expression on miR-509-3-5p expression. **H,** The effects of altered circMLLT10 expression on the level of GINS4 protein and the activation of Rac1/CDC4 and their downstream pathways were detected by western blot. **I&J**, Negative control (NC), wild-type (WT) or mutant (Mutant) of circMLLT10 sequences were respectively cloned into luciferase reporter plasmids. The relative luciferase activity was detected in MGC-803 and AGS cells. **K&L**, The effects of altered circMLLT10 and miR-509-3-5p expression on GINS4 mRNA and protein expression were detected by qRT-PCR and western blot. All data are presented as the mean ± SEM of three experiments. **P<0.01.

To identify circRNAs that are crucial for gastric cancer growth and progression, circRNA sequencing was performed to detect differentially expressed circRNAs in 6 paired fresh frozen gastric cancer tissues and normal gastric tissues, with 104 differentially expressed circRNAs identified with >2- or <0.5-fold changes (*P*<0.05) (**Figure 7A**). Bioinformatics analysis revealed that circMLLT10, which is significantly highly expressed in gastric cancer tissues (9.894-fold change, *P*=0.015), possesses binding sites for miR-509-3-5p. Therefore, we focused on circMLLT10, which is alternatively spliced from the MLLT10 gene at chr10:21713772-21735235, with an ultimate length of 1256 nucleotides; a schematic representation is shown in **Figure 7B**. Then, circMLLT10 was verified to be highly expressed in 81.97% (50/61) of fresh frozen gastric cancer tissues (**Figure 7C**), was negatively correlated with miR-509-3-5p expression (r=-0.374, *P*=0.003), and positively correlated with GINS4 expression (r=0.366, *P*=0.004) (**Figure 7D**), indicating that circMLLT10 may be involved in regulating miR-509-3-5p and GINS4 expression. In addition, the FISH results revealed that circMLLT10 and miR-509-3-5p were colocalized in the cytoplasm in MGC-803 cells and AGS cells (**Figure 7E**). All these results revealed that circMLLT10 interacts with miR-509-3-5p in gastric cancer.

To investigate the regulation of miR-509-3-5p by circMLLT10, three siRNAs (si-circ-1, si-circ-2, si-circ-3) (**Figure S3E**) targeting circMLLT10 and an overexpression vector (pEX3-circ) of circMLLT10 were generated. CircMLLT10 expression was significantly silenced by the three siRNAs in MGC-803 cells, while MLLT10 mRNA did not change (**Figure 7F**). Similarly, circMLLT10 was obviously overexpressed in AGS cells, but not MLLT10 mRNA (**Figure 7F**). Among the 3 si-RNAs, si-circ-1 exhibited the highest knockdown efficiency in MGC-803 cells. Then, we detected the effect of altered circMLLT10 expression on miR-509-3-5p expression, and found that knockdown of circMLLT10 increased miR-509-3-5p expression, and overexpression of circMLLT10 expression decreased miR-509-3-5p expression (**Figure 7G**). Further bioinformatics analysis revealed that circMLLT10 possesses two binding sites for miR-509-3-5p. Then, full length circMLLT10 sequences (WT) and circMLLT10 sequences with both mutant binding sites (Mutant) were constructed (**Figure S3F**). Luciferase reporter assay results indicated that miR-509-3-5p/inhibitor significantly increased the activity of WT vector but did not affect the activity of Mutant vector (**Figure 7I**); miR-509-3-5p/mimics substantially decreased the luciferase activity of WT vector but did not decrease that of Mutant vector (**Figure 7J**). In summary, these results demonstrated that circMLLT10 acts as a miR-509-3-5p sponge in gastric cancer.

### CircMLLT10 promotes cell growth and metastasis

**Figure 8:**
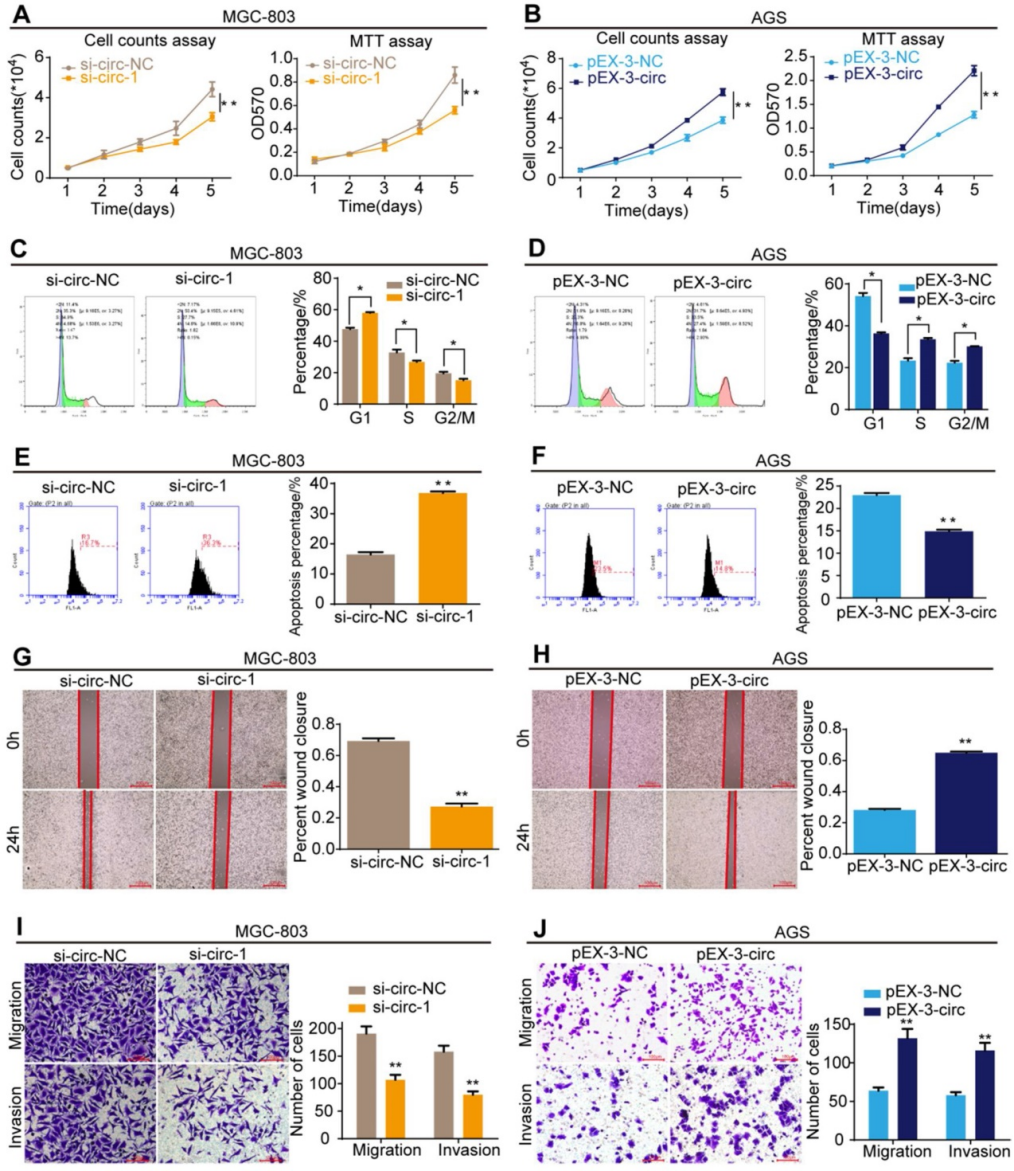
** CircMLLT10 promotes cell proliferation, cell cycle, migration and invasion, and suppresses cell apoptosis *in vitro*. A**, Cell counting and MTT assays for MGC-803 cells with si-circ-NC and si-circ-1. **B**, Cell counting and MTT assays for AGS cells with pEX-3-NC and pEX-3-circ. **C&D**, Flow cytometric analysis of the cell cycle in MGC-803 and AGS cells. **E&F**, Flow cytometric analysis of cell apoptosis in MGC-803 and AGS cell. **G&H**, Cell wound healing assays of MGC-803 and AGS cells with different levels of circMLLT10.** I&J,** Transwell migration and invasion assays of MGC-803 and AGS cells with different levels of circMLLT10. All data are presented as the mean ± SEM of three experiments. *P<0.05, **P< 0.01.

The effects of circMLLT10 on cell growth and metastasis were detected. Cell counting and MTT assays showed that down-regulation of circMLLT10 expression markedly decreased the proliferation rate of MGC-803 cells (**Figure 8A**), while up-regulation of circMLLT10 expression markedly increased that of AGS cells (**Figure 8B**). In cell cycle assays, decreased circMLLT10 induced G1 phase arrest (**Figure 8C**), and increased circMLLT10 promoted the G1/S phase transition (**Figure 8D**). In cell apoptosis assays, knockdown of circMLLT10 increased cell apoptosis in MGC-803 cells (**Figure 8E**), whereas overexpression of circMLLT10 decreased cell apoptosis in AGS cells (**Figure 8F**). In addition, cell wound healing and transwell migration and invasion assays indicated that knockdown of circMLLT10 decreased the ability of mobility, migration and invasion (**Figure 8G&I**), while overexpression of circMLLT10 enhanced the ability of mobility, migration and invasion (**Figure 8H&J**). In summary, circMLLT10 promoted cell proliferation, cell cycle and metastasis, and suppressed cell apoptosis in gastric cancer.

### CircMLLT10 promoted GINS4 expression and activated Rac1 and CDC42 through miR-509-3-5p

To verify the regulation of circMLLT10 on GINS4 expression, we tested that down-regulation of circMLLT10 significantly decreased GINS4 mRNA and protein expression, and up-regulation of circMLLT10 increased GINS4 mRNA and protein expression (**Figure 7H&K&L**), indicating that circMLLT10 promotes GINS4 expression. Furthermore, miR-509-3-5p/inhibitor counteracted the ability of circMLLT10 knockdown to decrease the expression of GINS4, while miR-509-3-5p/mimics attenuated the ability of circMLLT10 overexpression to enhance the expression of GINS4 (**Figure 7K&L**); showing that miR-509-3-5p reversed the ability of circMLLT10 to promote GINS4 expression.

To investigate whether circMLLT10 regulates the activation of Rac1 and CDC42, we detected that knockdown of circMLLT10 decreased the level of GTP-Rac1 and GTP-CDC42, also, the expression of pERK1/2, pAKT and pPTEN; while overexpression of circMLLT10 increased the level of GTP-Rac1 and GTP-CDC42, also, the expression of pERK1/2, pAKT and pPTEN (**Figure 7H**). In summary, we demonstrate that circMLLT10 promotes GINS4 expression through miR-509-3-5p, thereby activating Rac1 and CDC42 and their downstream pathways.

## Discussion

In the present study, we found that the novel gene GINS4 was closely correlated with the clinicopathological features of gastric cancer and promoted gastric cancer growth and progression. Mechanistically, GINS4 directly binds to and activates Rac1 and CDC42, thereby activating their downstream pathways. Furthermore, the novel circRNA-circMLLT10 was found to function as a ceRNA by harboring miR-509-3-5p, eliminating its suppression on GINS4 expression. Collectively, a novel circMLLT10/miR-509-3-5p/GINS4/Rac1/CDC42 axis was established in gastric cancer growth and progression.

GINS4 plays a pivotal role in chromosome replication initiation and elongation, and promotes the G1/S phase transition of the cell cycle 23. In this study, GINS4 expression was higher in gastric cancer tissues than in normal tissues, and correlated with poor differentiation and advanced T stage, N stage and disease stage. Additionally, gastric cancer patients with high GINS4 expression exhibited a low OS and DFS. These results indicated that GINS4 was closely correlated with gastric cancer development and progression, which was further supported by cell experiments *in vitro* and *in vivo*. Consistent with a recent study indicating that GINS4 expression was high in human bladder cancer tissues and that knockdown of GINS4 expression suppressed cancer cell growth 12, our findings suggested that GINS4 promoted cell growth and metastasis *in vitro* and* in vivo*. All these results demonstrated that GINS4 acted as a tumor oncogene and played key roles in gastric cancer growth and progression, and may function as a promising target for gastric cancer therapy and diagnosis.

To explore the downstream mechanisms underlying GINS4, GINS4 expression in MGC-803 cells was knocked-down, and cDNA arrays were performed to screen for altered genes. IPA revealed that GINS4 plays key roles in cell proliferation, cell cycle, apoptosis, migration and invasion of gastric cancer by Rac1 and CDC42 and their downstream signaling pathways: MAPK/ERK pathway, PI3K/AKT pathway and PTEN pathway. Rac1 and CDC42 have been widely shown to participate in cell proliferation, cell cycle and apoptosis, migration and invasion through a variety of signaling pathways 24-27. As main Rho GTPase family members 28, Rac1 and CDC42 function as molecular switches, cycling between two functional states, a GDP-bound, inactive state and a GTP-bound, active state. In the present study, pull-down and co-IP assays demonstrated that GINS4 directly binds to Rac1/CDC42 *in vivo* and *in vitro*. Furthermore, GTPase activation assays revealed that GINS4 enhanced the level of GTP-Rac1 and GTP-CDC42, demonstrating that GINS4 activates Rac1 and CDC42. In summary, we demonstrate that GINS4 activates Rac1 and CDC42 through directly binding to Rac1 and CDC42.

It has been reported that activated Rac1/CDC42 promotes cell proliferation through the mitogen-activated protein kinase kinase (MEK)/extracellular regulated protein kinases (ERK) cascade 29 and activates the Jun N-terminal kinase (JNK) pathway^30^. A recent study found that CDC42 expression correlates positively with Ki-67 expression in breast cancer 31. In addition, Rac1 promotes cell cycle progression through MAPK pathways, including the p38 32 and JNK pathways 33. Additionally, Rac1 also promotes cyclinD1 expression through the nuclear factor-κB (NF-κB) pathway 34. Moreover, activated Rac1/CDC42 has been reported to suppress cell apoptosis by activating the Raf-MEK-Erk cascade 35 or binding to and stabilizing Bcl-2 36. In this study, we detected that GINS4 increased the expression pERK1/2, pAKT and pPTEN, also the expression of cell proliferation-, cycle-, apoptosis- and metastasis-related proteins (Ki-67, Bcl-2, cyclinD1, N-Cadherin and Vimentin). Taken together, GINS4 plays key roles in cell proliferation, cell cycle, apoptosis, migration and invasion of gastric cancer through activating Rac1 and CDC42 and their downstream signaling pathways: MAPK/ERK pathway, PI3K/AKT pathway and PTEN pathway.

MiRNAs have been frequently reported to play key roles in the development and progression of various cancers 37, 38. Our previous studies revealed that miR-509-3-5p expression is markedly low in gastric cancer tissues and is negatively correlated with tumor differentiation, disease stage and T stage, implying that miR-509-3-5p acts as a tumor suppresser in gastric cancer 39. In addition, miR-509-3-5p suppressed cell migration and invasion and lymph node metastasis by transcriptionally inhibiting PODXL mRNA expression. Another report on human lung cancer indicated that miR-509-3-5p induced G2/M arrest and inhibited the proliferative ability of A549 cells by suppressing PLK1 expression 40. These reports verified that aberrant miR-509-3-5p expression plays a vital role in tumor progression. In the current study, our evidence specifically proves that miR-509-3-5p also suppresses GINS4 expression via directly binding to GINS4 mRNA, thereby inhibiting cell growth and metastasis. Interestingly, a recent study reported that miR-370 also negatively modulated GINS4 expression 12. It would be meaningful to further investigate more regulators of GINS4 expression, including transcription factors, miRNAs, long non-coding RNAs, and circRNAs, and how these regulators interact with each other and contribute to gastric cancer growth and progression^41^, ^42^.

As a new popular focus in the field of non-coding RNA, circRNAs play important roles in cancer development and progression 43-46. Indeed, an increasing amount of evidence suggests that circRNAs may function as miRNA sponges to negatively modulate miRNA activity, eliminating the inhibitory effect on target genes, which is termed the ceRNA hypothesis. Chen et al. found that circPVT1 acted as a ceRNA, sponging the miR-125 family (miR-125a, miR-125b) and promoting gastric cancer cell proliferation 47. In addition, circCCDC66 was found to promote colon cancer proliferation, migration and metastasis* in vitro* and *in vivo* 14. Mechanistic studies revealed that circCCDC66 plays an oncogenic role by sponging miR-33b and miR-93, protecting MYC mRNA from degradation. Additionally, Chen et al. reported that by sponging miR-29b, circRNA_100290 was up-regulated in OSCC, co-expressed with CDK6, and functioned as a ceRNA to enhance CDK6 expression 15. Another study revealed that circMTO1 acted as a sponge for miR-9, promoting p21 expression and inhibiting cell proliferation and metastasis 48. In our current study, circMLLT10 was found to be highly expressed in gastric cancer tissues. We identified that circMLLT10 functions as a miRNA sponge for miR-509-3-5p to relieve its suppression on GINS4 expression, thereby activating Rac1 and CDC42 and their downstream pathways. First, the level of circMLLT10 expression correlated positively with GINS4 expression and negatively with miR-509-3-5p expression in 61 fresh frozen gastric cancer tissues. Second, decrease in circMLLT10 expression increased miR-509-3-5p expression and decreased GINS4 mRNA and protein expression in gastric cancer cells, whereas overexpression of circMLLT10 had the opposite effects. Third, bioinformatics predictions and luciferase reporter assays indicated that circMLLT10 recruits and directly interacts with miR-509-3-5p, and miR-509-3-5p also directly binds to the GINS4 mRNA 3'UTR. Fourth, circMLLT10 and miR-509-3-5p co-localized in the cytoplasm. Fifth, decreases in miR-509-3-5p expression reversed the reduced expression of GINS4 caused by circMLLT10 down-regulation, and miR-509-3-5p overexpression decreased GINS4 expression, which was increased by circMLLT10 overexpression. Lastly, circMLLT10 activated Rac1 and CDC42 and their downstream pathways. To our knowledge, the majority of circRNAs is present in a low abundance level and may not function substantially in cell processes. Therefore, the discovery of circMLLT10, a novel ceRNA, would be enlightening. In addition, based on the knowledge that one circRNA may contain multiple miRNA binding sites and that one miRNA can also bind to multiple circRNAs, circRNAs and miRNAs may engage in crosstalk in biological processes. Accordingly, more endeavors and further studies are needed to reveal the functions and mechanisms of circRNAs in cancer progression.

## Conclusions

In summary, our current study provides distinct insights into the roles of the novel GINS4 axis in gastric cancer growth and progression. GINS4 is a promising biomarker for early diagnosis and molecular target for therapeutic modalities in gastric cancer.

## Supplementary Material

Supplementary materials and methods, figures, tables.

## Figures and Tables

**Table 1:** Correlation between GINS4 expression and clinicopathological parameters in gastric cancer (n=54).

Parameters	Category	No.	GINS4 expression			*χ^2^*	*P*
			Negative	Weak positive	Strong positive		
Age							
	<65	26	3	7	16	1.311	0.519
	≥65	28	6	5	17		
Gender							
	Male	38	5	7	26	2.859	0.239
	Female	16	4	5	7		
T stage							
	T2+T3	26	5(19.23)	10(38.46)	11(42.31)	9.597	**0.008**
	T4	28	4(14.29)	2(7.14)	22(78.57)		
N stage							
	N0+N1	23	4(17.39)	9(39.13)	10(43.48)	7.324	**0.026**
	N2+N3	31	5(16.13)	3(9.68)	23(74.19)		
UICC stage							
	II	17	4(23.53)	7(41.18)	6(35.29)	7.418	**0.024**
	III	37	5(13.51)	5(13.51)	27(72.97)		
Nerve invasion							
	Yes	29	6	8	15	2.355	0.308
	No	25	3	4	18		
Vessel invasion							
	Yes	29	5	7	17	0.180	0.914
	No	25	4	5	16		
Differentiation							
	Well	3	3(100)	0	0	11.210	**0.003**
	Moderate+Poor	51	6(11.76)	12(25.49)	33(62.75)		
Tumor size							
	≤3cm	23	4	2	17	4.774	0.092
	>3cm	31	5	10	16		
Tumor and normal							
	Tumor	54	9	12	33	15.157	**0.001**
	Normal	54	17	24	13		

**Table 2:** Univariate and multivariate analysis for overall survival (OS) and disease-free survival (DFS) in gastric cancer

Parameters	No.	OS				DFS			
		Univariate analysis		Multivariate analysis		Univariate analysis		Multivariate analysis	
		*χ^2^*	*P*	HR(95%CI)	*P*	*χ^2^*	*P*	HR(95%CI)	*P*
Age		1.899	0.168			1.130	0.288		
<65	26								
≥65	28								
Gender		0.039	0.844			0.000	0.984		
Male	38								
Female	16								
T stage		10.011	**0.002**	1.943(0.709-5.327)	0.197	9.298	**0.002**	1.609(0.633-4.089)	0.318
T2+T3	26								
T4	28								
N stage		7.655	**0.006**	1.176(0.462-2.998)	0.734	8.500	**0.004**	1.088(0.434-2.727)	0.857
N0+N1	23								
N2+N3	31								
UICC stage		9.714	**0.002**	1.658(0.382-7.196)	0.500	11.367	**0.001**	1.976(0.491-7.945)	0.338
II	17								
III	37								
Nerve invasion		0.229	0.632			0.053	0.817		
Yes	29								
No	25								
Vessel invasion		0.214	0.643			0.072	0.789		
Yes	29								
No	25								
Differentiation		0.360	0.548			0.409	0.522		
Well	3								
Moderate+Poor	51								
Tumor size		3.337	0.068			1.032	0.310		
≤3cm	23								
>3cm	31								
GINS4 expression		7.720	**0.005**	2.026(0.893-4.594)	0.091	7.073	**0.008**	1.820(0.855-3.876)	0.120
Weak or negative positive	21								
Strong positive	33								

HR: hazard ratio; CI: confidence interval.

## Abbreviations

qRT-PCR: Real-time quantitative polymerase chain reaction
TMA: Tissue microarray
CircRNA: Circular RNA
TCGA: The Cancer Genome Atlas
ORC: origin recognition complex
CDT1: CDCl0-dependent transcript 1
pre-RC: pre-replicative complex
OSCC: oral squamous cell carcinoma
ceRNA: Competing endogenous RNA
miRNA: MicroRNA
MREs: microRNA response elements
UICC: the Union for International Cancer Control
FBS: fetal bovine serum
DIG: digoxigenin
PLB: passive lysis buffer
co-IP: Co-immunoprecipitation assay
GST: Glutathione S-transferase
FISH: Fluorescence *in situ* hybridization
IPA: ingenuity pathway analysis
ISH: *In situ* hybridization
OS: overall survival
DFS: disease-free survival
3'UTR: 3′ -untranslated region
MEK: mitogen-activated protein kinase kinase
ERK: extracellular regulated protein kinases
JNK: Jun N-terminal kinase
NF-κB: nuclear factor-κB
