# Supplementary material for: The novel GINS4 axis promotes gastric cancer growth and progression by activating Rac1 and CDC42
Source: Theranostics. 2019 Oct 21;9(26):8294–311. doi: 10.7150/thno.36256 (PMC6857050; doi:10.7150/thno.36256)
Supplement: Supplementary file 1 — Supplementary materials and methods, figures, tables. [file thnov09p8294s1.pdf]

Additional files:

Figure S1.

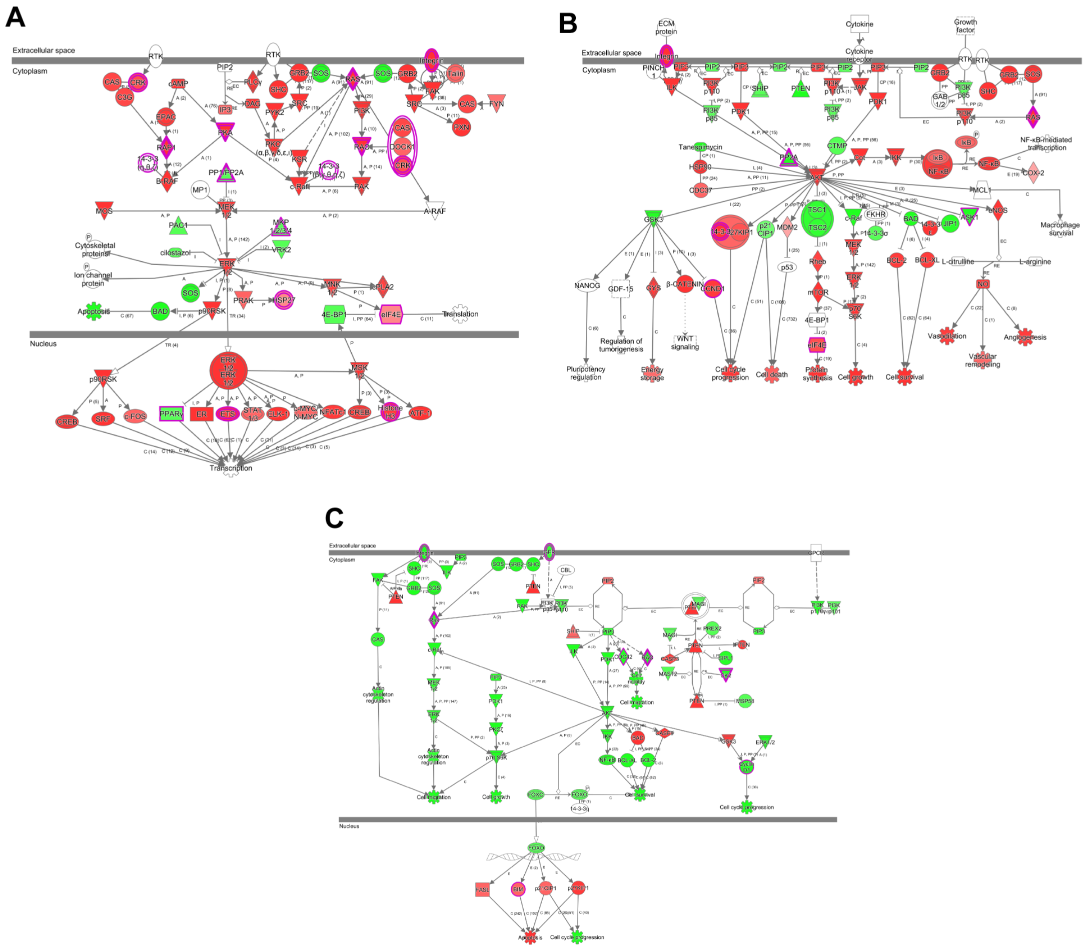

Figure S1. IPA showed the network through which GINS4 may promote gastric cancer growth and metastasis through 3 pathways. IPA revealed that GINS4 may participate in the MAPK/ERK (**A**), PI3K/AKT (**B**) and PTEN (**C**) pathways in gastric cancer growth and metastasis.

Figure S2

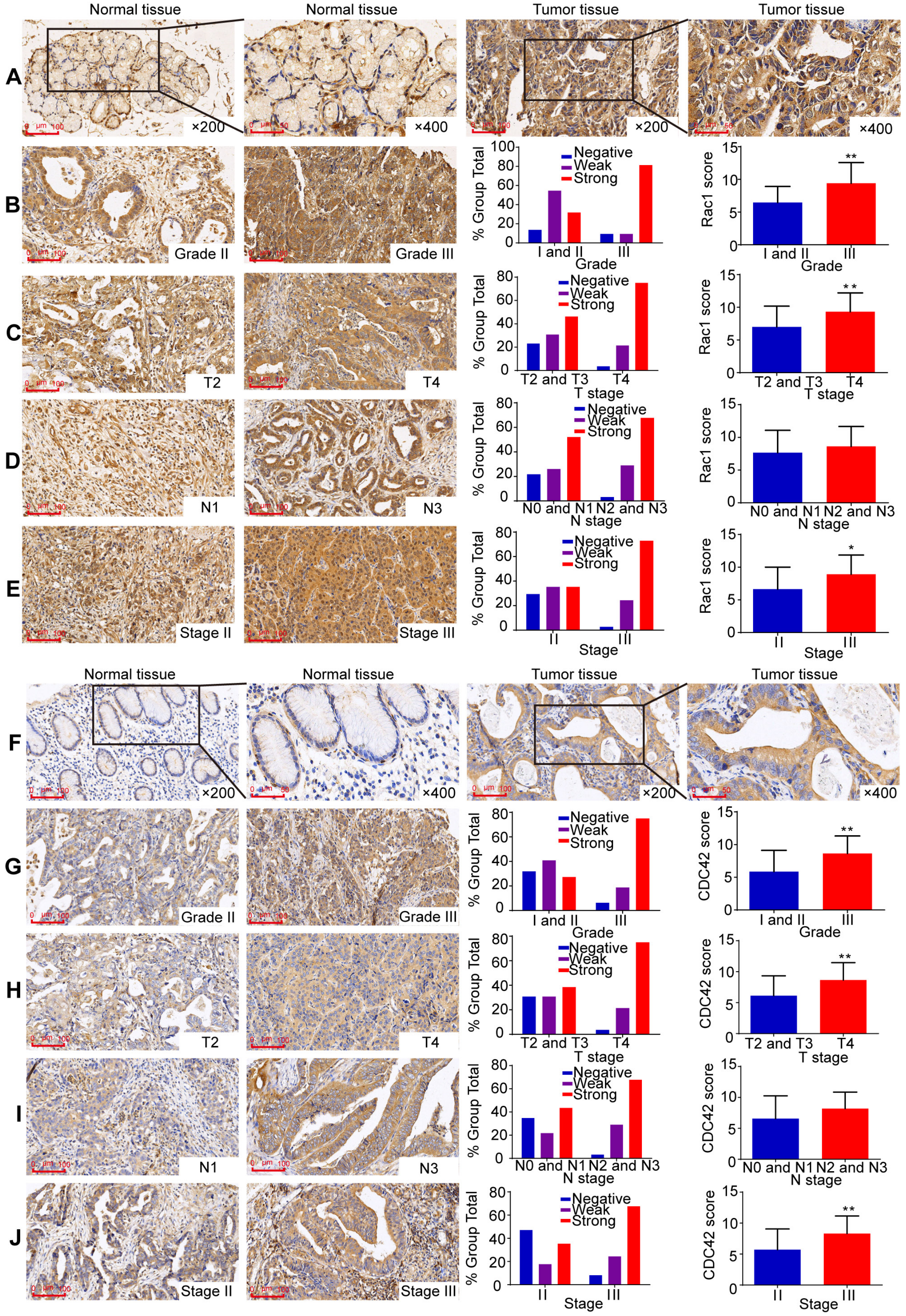

Figure S2. Rac1 and CDC42 protein expression in gastric cancer tissues and the correlation with clinicopathological features. The gastric cancer TMA was immunostained with specific anti-Rac1 and anti-CDC42 antibodies. **A**, Representative

images of Rac1 protein expression in normal gastric tissues and matched gastric cancer tissues. **B-E**, Difference in Rac1 expression between grade I vs grades II and III (**B**); T4 vs T2 and T3 (**C**); N0 and N1 vs N2 and N3 (**D**); and stage III vs stage II (**E**). F, Representative images of CDC42 protein expression in normal gastric tissues and matched gastric cancer tissues. **G-J**, Difference in CDC42 expression between grade I vs grades II and III (**G**); T4 vs T2 and T3 (**H**); N0 and N1 vs N2 and N3 (**I**); and stage III vs stage II (**J**). **P*<0.05, ***P*<0.01.

Figure S3.

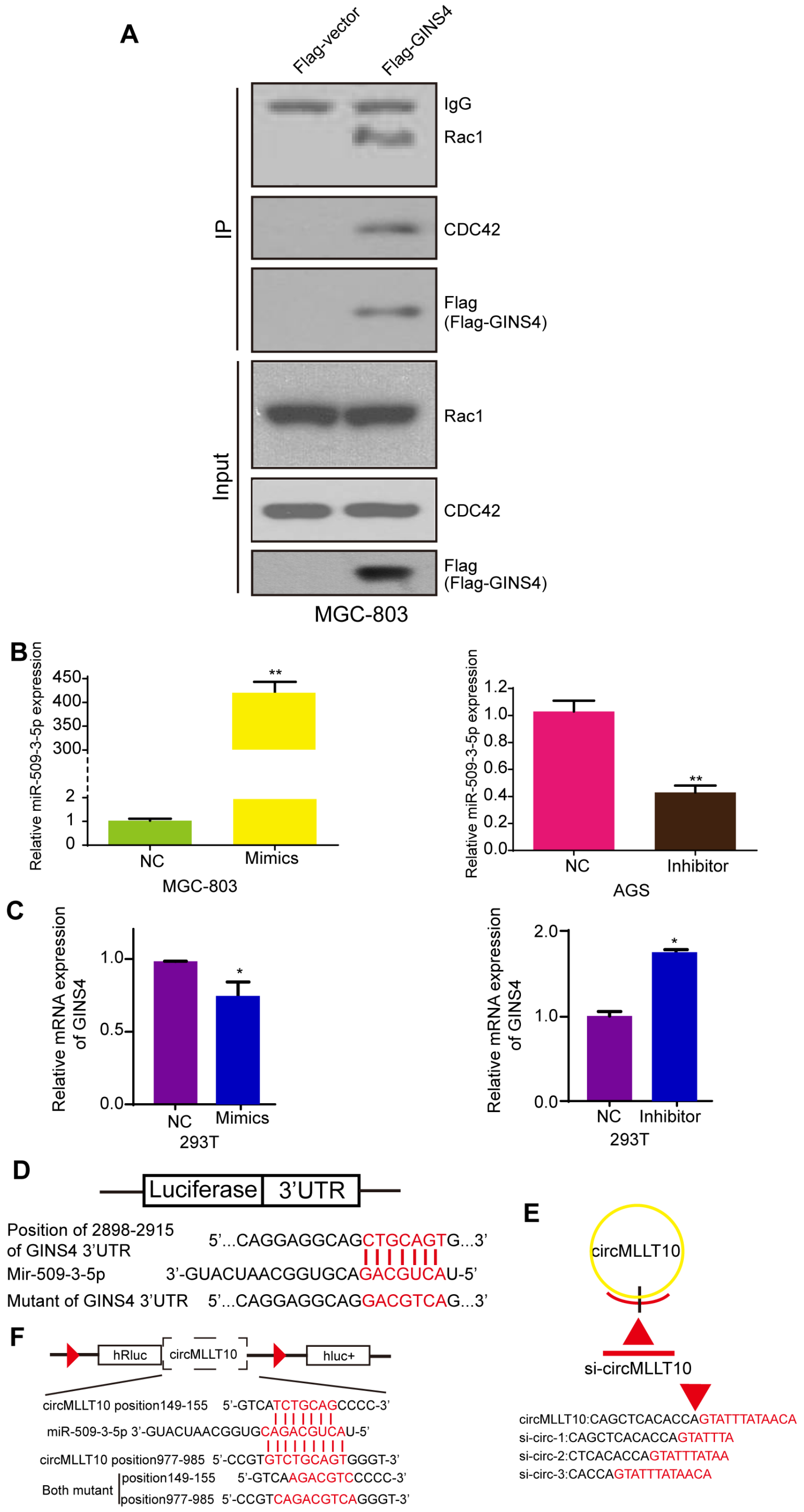

Figure S3 co-IP assays in MGC-803 cells and the effects of miR-509-3-5p on the expression of GINS4. A, For co-IP assay, flag-vector or Flag-GINS4 plasmids were transfected into MGC-803 cells. Endogenous Rac1 and CDC42 were

immunoprecipitated with an anti-Flag antibody. **B,** The efficiencies of miR-509-3-5p/mimics and miR-509-3-5p/inhibitor. **C,** The effects of miR-509-3-5p on the expression of GINS4. **D,** Potential binding site for miR-509-3-5p on the wild-type (WT) and mutant (Mutant) 3'UTR of GINS4 mRNA. **E,** The illustration of three si-RNA targeting different sites of circMLLT10. **F,** Schematic representation of 2 potential miR-509-3-5p binding sites in circMLLT10 and both mutant binding sites. All data are presented as the mean ± SEM of three experiments. *P<0.05, **P< 0.01.

Figure S4

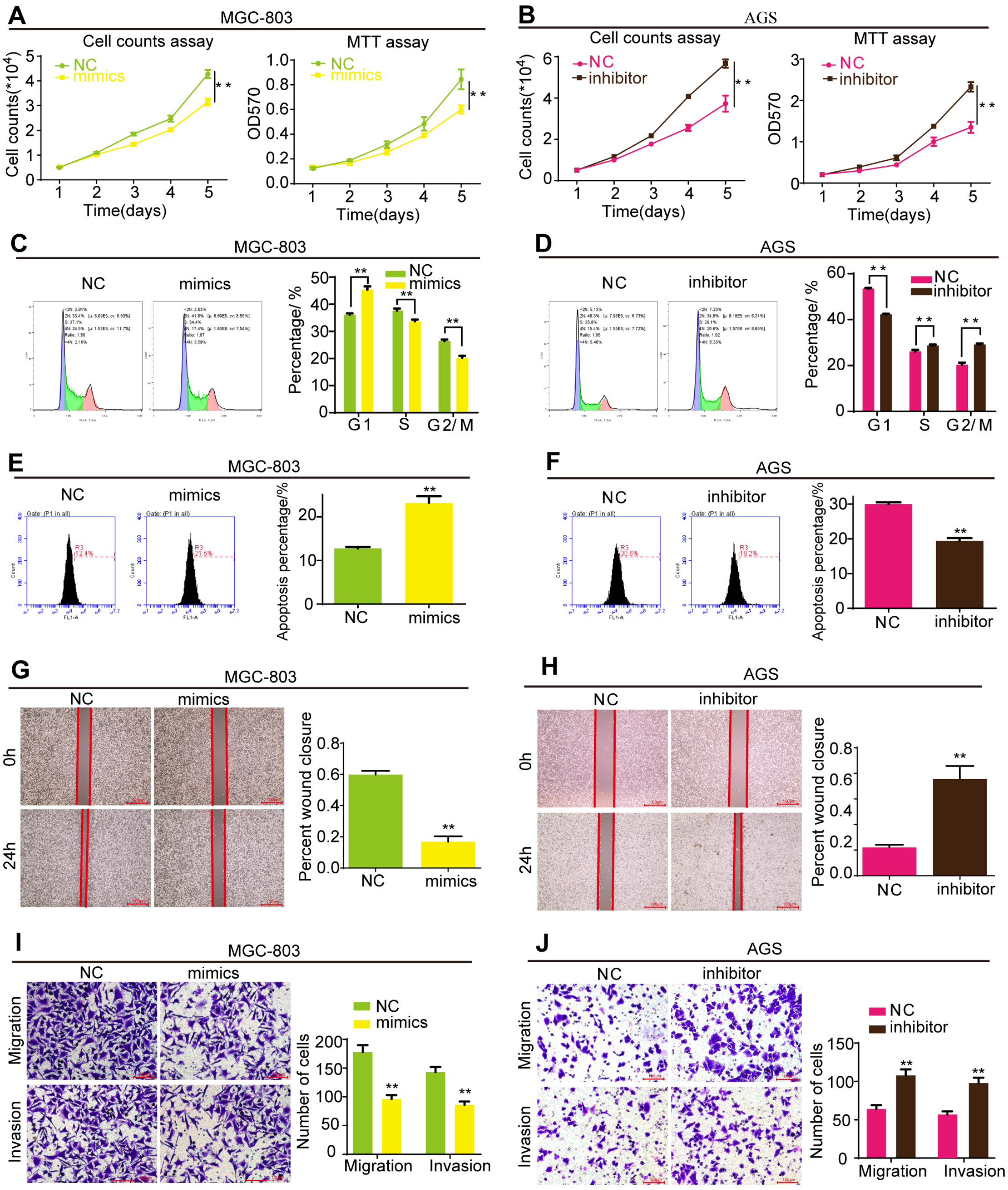

Figure S4 MiR-509-3-5p suppresses cell proliferation, cell cycle, migration and invasion, and promotes cell apoptosis *in vitro*. A, Cell counting and MTT assays for MGC-803 cells with miR-509-3-5p/NC and miR-509-3-5p/mimics. **B**, Cell counting and MTT assays for AGS cells with miR-509-3-5p/NC and miR-509-3-5p/inhibitor. **C&D**, Flow cytometric analysis of the cell cycle in MGC-803 and AGS cells. **E&F**, Flow cytometric analysis of cell apoptosis in MGC-803 and AGS cell. **G&H**, Cell wound healing assays of MGC-803 and AGS cells with different levels of

miR-509-3-5p. **I&J,** Transwell migration and invasion assays of MGC-803 and AGS cells with different levels of miR-509-3-5p. All data are presented as the mean ± SEM of three experiments. *P<0.05, **P< 0.01.

Table S1. Correlation between Rac1 expression and clinicopathological parameters in gastric cancer (n=54)

Parameters	Category	No.	Rac1 expression			χ^2	P
			Negative	Weak positive	Strong positive		
Age						3.065	0.216
	<65	26	2	5	19		
	≥65	28	4	10	14		
Gender						0.110	0.946
	Male	38	4	11	23		
	Female	16	2	4	10		
T stage						5.550	**0.018**
	T2+T3	26	5	9	12		
	T4	28	1	6	21		
N stage						4.822	0.090
	N0+N1	23	5	6	12		
	N2+N3	31	1	9	21		
UICC stage						8.198	**0.017**
	II	17	5	6	6		
	III	37	1	9	27		
Nerve invasion						1.260	0.533
	Yes	29	2	9	18		
	No	25	4	6	15		
Vessel invasion						3.373	0.185
	Yes	29	3	11	15		
	No	25	3	4	18		
Differentiation						15.562	**0.000**
	Well+ Moderate	22	3	12	7		
	Poor	32	3	3	26		
Tumor size						2.549	0.280
	≤3cm	23	1	8	14		
	>3cm	31	5	7	19		
Tumor and normal							
	Tumor	54	6	15	33	29.387	**0.000**
	Normal	54	16	32	6		

Table S2. Correlation between CDC42 expression and clinicopathological parameters in gastric cancer (n=54)

Parameters	Category	No.	CDC42 expression			χ^2	P
			Negative	Weak positive	Strong positive		
Age						8.456	0.015
	<65	26	3	3	20		
	≥65	28	6	11	11		
Gender						0.071	0.965
	Male	38	6	10	22		
	Female	16	3	4	9		
T stage						10.400	**0.006**
	T2+T3	26	8	8	10		
	T4	28	1	6	21		
N stage						10.157	**0.006**
	N0+N1	23	8	5	10		
	N2+N3	31	1	9	21		
UICC stage						5.148	**0.023**
	II	17	6	4	7		
	III	37	3	10	24		
Nerve invasion						8.654	**0.013**
	Yes	29	1	9	19		
	No	25	8	5	12		
Vessel invasion						2.762	0.251
	Yes	29	5	10	14		
	No	25	4	4	17		
Differentiation						10.523	**0.001**
	Well+ Moderate	22	7	8	7		
	Poor	32	2	6	24		
Tumor size						0.243	0.662
	≤3cm	23	5	5	13		
	>3cm	31	4	9	18		
Tumor and normal							
	Tumor	54	9	14	31	12.573	**0.002**
	Normal	54	14	27	13		

Table S3. The sequences of primers for qRT-PCR.

GINS4	Sense	TATGGGCTGCGAGAGTAATC
	Antisense	CCTGGGTAACAAGAGGGAAA
circMLLT10	Sense	ACTTTCTGACCAGCAACGACA
	Antisense	GCAAATGCCCAGAAGACTGC
MLLT10	Sense	GAGTGGAGTTCAGCAGGTCAATGG
	Antisense	CGGCAGGAATGGTGGAAGTTACAG
Rac1	Sense	GCCATCAAGTGTGTGGTGGT
	Antisense	CCATCTACCATAACATTGGCAGAA
CDC42	Sense	CGTGACCTGAAGGCTGTCAA
	Antisense	GGCTCTTCTTCGGTTCTGGA
Ki-67	Sense	AGAGAGCCAGCAGGAGATGG
	Antisense	GGCCTCTTCCTTAGGCGTTC
BCL-2	Sense	CATGTGTGTGGAGAGCGTCA
	Antisense	CAGGTGCCGGTTCAGGTACT
cyclinD1	Sense	CAGAAGTGCGAGGAGGAGGT
	Antisense	TAGAGGCCACGAACATGCAA
GAPDH	Sense	GGGAAGGTGAAGGTCGGAGT
	Antisense	GGGGTCATTGATGGCAACA
miR-509-3-5p	stem-loop	GTCGTATCCAGTGCAGGGTCCGAGGTATTCGCACTGGATACGACCATGAT
	Sense	GCGTACTGCAGACGTGGCA
	Antisense	AGTGCAGGGTCCGAGGTATT
U6	stem-loop	CTCAACTGGTGTCGTGGAGTCGGCAATTCAGTTGAGAAAAATAT
	Sense	CAAGGATGACACGCAAA
	Antisense	TCAACTGGTGTCGTGG

Supplementary materials and methods:

Transfection, oligonucleotides and plasmids

To manipulate circMLLT10 and miR-509-3-5p expression, oligonucleotides and plasmids were designed and synthesized. The following siRNAs targeting circMLLT10 were synthesized by RiboBio (Guangzhou, China): si-circ-1 target, 5'-CAGCTCACACCAGTATTTA-3'; si-circ-2 target, 5'-CTCACACCAGTATTTATAA-3'; and si-circ-3 target, 5'-CACACCAGTATTTATAACA-3'. Full length circMLLT10 was cloned into the pEX-3 (Shanghai GenePharma Co., Ltd.) overexpression vector. The mimics, inhibitor and negative controls for hsa-miR-509-3-5p were purchased from RiBoBio (Guangzhou, China). The oligonucleotides and plasmids were transfected into cells using Lipofectamine^{TM} 2000 (Invitrogen) according to the manufacturer's instructions. The GINS4, luciferase and Flag sequences were cloned into the lentiviral vector (pLVX-IRES-puro). The shRNA-GINS4 sequences were as follows: 5'-ACACAGAGTCCTATCTGAA-3' (target), 5'-CCTATTTCCCATGATTCCTTCATA-3' (sense), and 5'-GTAATACGGTTATCCACGCG-3' (antisense). Stable cell lines were selected and purified in puromycin-containing medium (Sigma-Aldrich, St. Louis, MO).

The cancer genome atlas (TCGA) database, Oncomine database and Kaplan-Meier Plotter

Following approval of this project, the gastric cancer data from TCGA database (https://cancergenome.nih.gov) was downloaded. There were 443 samples with available data in TCGA database, including 416 samples with mRNA chip or RNA-seq data; among these samples, 32 paired samples possessed RNA-seq v2 and pathological data. The 32 paired samples were used for RNA-seq analysis, including 4 Stage I, 18 Stage II, 6 Stage III and 4 Stage IV samples. Differentially expressed genes between gastric cancer tissues and paired normal gastric tissues were screened bioinformatically. After filtration and standardization, significantly differentially expressed genes were found in gastric cancer tissues. The Oncomine database (https://www.oncomine.org) was used to acquire GINS4 mRNA data in gastric cancer

and normal gastric mucosae. GINS4 mRNA expression was evaluated in 90 paired gastric cancer and normal tissues. The Kaplan-Meier Plotter (http://kmplot.com) tool was used to find the effect of GINS4 expression on survival in gastric cancer patients. Of the 876 available patients, 652 patients presented with low GINS4 expression, and 224 patients had high GINS4 expression.

cDNA array and Ingenuity Pathway Analysis (IPA)

Total RNA was extracted and analyzed by an Agilent 2100 bioanalyzer, and then, amplified RNAs (aRNAs) were prepared by a GeneChip 3'IVT Express Kit (Affymetrix, 901838). After fragment purification, aRNAs were hybridized to chip probes. After washing and dyeing, pictures and raw data were scanned and acquired. Differentially expressed genes were screened, and clustering graphs were constructed. To analyze the validated target genes and biological networks in the cDNA array, IPA was performed. All enrichment analyses were statistically identified using IPA. The data were imported into IPA software for canonical pathway, upstream, disease and function, regulator effect and interaction network analyses. Subsequently, a P-value or P-value range for each molecule, pathway or network, suggestive of importance, was determined.

Cell counting assay and MTT assay

Cells were seeded into 96-well plates with 2000 cells per well and then cultured at 37 °C in a humidified atmosphere with 5% CO_2. The cells were counted with a Celigo® Image Cytometer (Nexcelom, USA) every 24 h for 5 days. For the MTT assay, 2000 cells per well were seeded into 96-well plates and cultured in the same condition. After culturing for 24 h, 10 μl of MTT solution (Beyotime Biotechnology, Jiangsu, China) was added to the 96-well plates. After incubating for 4 h, formazan solution was added into the above wells and incubated at 37 °C for 4 h. Absorbance was measured at 570 nm. MTT assays were performed every 24 h for 5 days. All experiments were executed in triplicate.

Flow cytometric analysis

For cell cycle assays, cells were collected and fixed in precooled 70% ethanol overnight. Cells were washed in 1 ml of precooled PBS and then stained with 10 μl of

RNase A and 25 μl of propidium iodide (PI) staining solution in 500 μl of dye buffer at room temperature for 30 min. Then, the cell cycle was detected by flow cytometry (BD Acuuri C6, USA) and analyzed by FlowJo software. Cell apoptosis was analyzed by an Annexin V Apoptosis Detection Kit (FITC) (eBioscience, 88-8005, USA). Cells were treated with trypsin without EDTA and collected. After washing with precooled PBS and diluting in 100 μl of binding buffer, 5 μL of fluorochrome-conjugated Annexin V was added into 100 μL of cell suspension and incubated for 15 min at room temperature. After centrifugation and resuspension in 200 μl of binding buffer, 5 μL of PI was added. Then, the cell apoptosis state was detected and analyzed by flow cytometry.

Cell wound healing and transwell assays

For cell wound healing assay, the cells were cultured to full confluence in 6-well plates. Next, the cells were scratched in the center of the well with a 20 μl micropipette tip. Subsequently, the cells were incubated with serum-free medium. Representative images were captured at 0 h and 24 h after injury. The ability of migration is calculated by the width of wound healing compared with baseline values.

For transwell assay, 2×10^4 cells in 200 μl serum-free medium were planted in the upper chamber (8.0 μm pore, Corning, USA) with (invasion) or without (migration) Matrigel (BD Bioscience, USA). 600μl RPIM medium with 10% FBS was added into the lower chamber. After culturing for 24 h, the upper chambers were fixed with 4% polymethanol for 30 min and then stained with 0.1% crystal violet for 30 min. Finally, the cells that migrated or invaded to the opposite side of the upper chambers were captured. Five random fields were selected to calculate cells.

RNA extraction and quantitative real-time PCR (qRT-PCR)

Total RNA was extracted from gastric cancer cell lines and tissues using TRIzol (TaKaRa, Shiga, Japan) according to the manufacturer's instructions. RNA was reverse transcribed into cDNA using a PrimeScript RT reagent kit (TaKaRa, Shiga, Japan). RNA expression was quantified by qRT-PCR with SYBR Premix Ex Taq™ (TaKaRa, Shiga, Japan) on a ViiA 7 Fast Real-Time PCR system (Applied Biosystems, NY, USA). GAPDH or U6 were used as internal controls for circRNA, mRNA and

miRNA. The ΔΔCt method was utilized to evaluate relative expression levels. The specific primers were provided in Table S3.

Protein extraction and western blotting

Total protein was separated and extracted from gastric cancer cells and tissues using radioimmunoprecipitation assay (RIPA) buffer with 1% protease inhibitor phenylmethanesulfony fluoride (Beyotime Biotechnology, Jiangsu, China). Protein concentrations were measured using a BCA protein assay kit (Beyotime Biotechnology, Jiangsu, China) according to the manufacturer's instructions. SDS-polyacrylamide gel electrophoresis (SDS-PAGE) was performed with 30 μg of total protein for each sample. Then, the protein was transferred onto polyvinylidene fluoride (PVDF) membranes (Millipore, MA, USA). Thereafter, the membranes were blocked in 5% non-fat milk at room temperature for 1.5 h, followed by incubation with appropriate primary antibody at 4 °C overnight. After washing with TBST buffer, the secondary antibodies were incubated at room temperature for 1.5 h. Protein bands were visualized by ECL chemiluminescent reagent (Millipore, MA, USA). The following antibodies were used: GINS4 (1:1000, Sigma, USA); Rac1 (1:10000, Abcam, USA); CDC42 (1:10000, Abcam, USA); Ki-67 (1:5000, Abcam, USA); Bcl-2 (1:1000, Abcam, USA); cyclinD1 (1:10000, Abcam, USA); GAPDH (1:1000; Cell Signaling Technology, USA); and β-tubulin (1:1000, Abcam, USA).

Immunohistochemical analysis (IHC)

After dewaxing with xylene and rinsing with ethanol, antigen retrieval was conducted by boiling the gastric cancer TMA for 5 min in 10 mM sodium-citrate buffer and blocking endogenous peroxidase activity with 3% hydrogen peroxide for 10 min. Thereafter, the TMA was incubated with the following primary antibodies at 4 °C overnight: GINS4 (1:500, Sigma, USA), Rac1 (1:800, Abcam, USA), CDC42 (1:800, Abcam, USA), KI-67 (1:300, Abcam, USA), Bcl-2 (1:500, Abcam, USA), and cyclinD1 (1:500, Abcam, USA). After incubating with secondary antibody at room temperature for 30 min, the TMA was then stained with DAB and hematoxylin, dehydrated and covered. The TMA staining scores were evaluated by two independent observers blinded to the clinicopathological data. The staining scores

were based on two indicators: the proportion of positively stained cells and the staining intensity. The proportion of positively stained cells was evaluated with five scoring levels: 0, <10%; 1, 10–25%; 2, 25–50%; 3, 50–75%; and 4, >75%. The staining intensity was scored with the following point system: 0 (no staining), 1 (yellow), 2 (yellow-brown) and 3 (dark brown). The products of the above two indicators were considered the total score. The total scores were relatively divided into three grades, ≤3 scores, >3 - ≤6 scores, and >6 scores, which corresponded with negative, weak positive and strong positive staining, respectively.
